# Centromere-size reduction and chromatin state dynamics following intergenomic hybridization in cotton

**DOI:** 10.1371/journal.pgen.1011689

**Published:** 2025-05-02

**Authors:** Jinlei Han, Guanjing Hu, Yan Dai, Xin Zhang, Jingjing Tian, Jialiang Zhou, Xinqi Xu, Qi Chen, Xiaobing Kou, Lei Xu, Xinyu Wu, Ziying Sun, Jiahui Geng, Lin Li, Chenyu Qiu, Teame Gereziher Mehari, Baohua Wang, Hui Zhang, Xinlian Shen, Zhenzhen Xu, Jonathan F. Wendel, Kai Wang

**Affiliations:** 1 School of Life Sciences, Nantong University, Nantong, China; 2 Department of Ecology, Evolution, and Organismal Biology, Iowa State University, Ames, Iowa, United States of America; 3 Shenzhen Branch, Guangdong Laboratory of Lingnan Modern Agriculture, Key Laboratory of Synthetic Biology, Ministry of Agriculture and Rural Affairs, Agricultural Genomics Institute at Shenzhen, Chinese Academy of Agricultural Sciences, Shenzhen, China; 4 Key Laboratory of Cotton and Rapeseed (Nanjing), Ministry of Agriculture and Rural Affairs, The Institute of Industrial Crops, Jiangsu Academy of Agricultural Sciences, Nanjing, China; University of Cambridge, UNITED KINGDOM OF GREAT BRITAIN AND NORTHERN IRELAND

## Abstract

Centromeres are pivotal for accurate chromosome segregation, yet their regulation and evolutionary dynamics remain poorly understood. Here, we investigate centromeres of the diploid species *Gossypium anomalum* (Ga, B-genome) that were transferred into tetraploid cotton *G. hirsutum* (Gh, AD-genome) as either an additional or integrated chromosome, as well as in synthetic allohexaploid (AABBDD) lines. We demonstrate consistent size reduction for all Ga centromeres in the Gh background. Histone modification profiling across 10 marks revealed heightened levels of both active and repressive chromatin marks within the Ga centromeres when transferred into the Gh background, particularly for H3K36me2. The centromeric histone modification perturbation extended into pericentromeric regions, with variable CENH3-binding domains consistently exhibiting a more pronounced increase in histone modification levels compared to stable centromere regions, highlighting the role of histone modification elevation in centromere dynamics. In addition, we observed enhanced chromatin accessibility and the presence of non-B-form DNA motifs, such as A-phased DNA repeats within stable centromere domains that are correlated with centromere stability. Hi-C analysis reveals a reorganized 3D chromatin architecture within the introgression line centromeres, including the formation of new topologically associating domains linked to H3K36me2 dynamics, emphasizing the importance of H3K36me2 in centromere organization. Together, these findings elucidate epigenetic mechanisms underlying centromere composition following intergenomic hybridization and allopolyploid formation, offering insights into centromere evolution in plants and its myriad epigenetic and potentially functional dimensions.

## Author summary

Centromeres are crucial for ensuring accurate chromosome segregation during cell division, yet their adaptability and regulatory mechanisms are not well understood. In cotton species, hybridization can lead to significant alterations in centromere characteristics, raising important questions about the underlying processes driving these adaptations. In our study, we focus on the centromeres of *Gossypium anomalum* when integrated into the *Gossypium hirsutum* genome, revealing a consistent reduction in centromere size. Our analysis uncovers substantial changes in histone modifications and chromatin architecture, particularly with elevated levels of H3K36me2 and enhanced chromatin accessibility. Additionally, we document alterations in topologically associating domains that are linked to these histone modification dynamics. These epigenetic changes appear to play a critical role in centromere stability and functionality, providing insights into how centromeres adapt during intergenomic hybridization and allopolyploid formation. This research enhances our understanding of centromere dynamics in plants, shedding light on the complex interplay of epigenetic factors that influence centromere adaptation and stability.

## Introduction

Centromeres are specialized chromosomal regions that orchestrate chromosome segregation during cell division, playing a crucial role in maintaining genomic stability [1–3]. Despite their indispensable role, centromeres remain enigmatic due to their complex sequence composition and dynamic epigenetic regulation. Plant centromeres are typically characterized by large arrays of species-specific satellite repeats and long-terminal repeat (LTR) retrotransposons, which envelop CENH3 nucleosomes [4–6]. CENH3, a centromere-specific histone H3 variant (known as CENP-A in humans and Cse4 in budding yeast), is a conserved feature of active centromeres across eukaryotes and is central to the assembly of the kinetochore [7,8], the protein structure that mediates chromosome-spindle apparatus attachment during cell division.

Centromeres in higher eukaryotes, defined by the presence of CENH3, typically span several kilobases to megabases, with sizes varying wildly across species. The total centromere size is positively correlated with genome size, and individual centromeres within a species tend to be uniform in size [9,10]. For example, while chromosome sizes in chicken can vary by up to 15-fold, their centromeres maintain similar sizes [11,12]. This raises an interesting question: how do centromeres change in size during interspecific hybridization between species with distinct centromere sizes? For instance, the oat genome (2n = 6x = 42) has an average centromere size of ~6.9 Mb [13], larger than that of maize (average size 2.2 Mb; 2n = 2x = 20) [14]. A study of oat-maize addition lines (where one maize chromosome is added to the oat background) revealed that maize centromeres expanded at least two-fold to size-equilibrate with oat centromeres [15], suggesting rapid centromere size equilibration. Additionally, recent research in *Brachypodium* species revealed that, following polyploidization, centromere size and sequence composition undergo changes as diploid genomes merge into a single nucleus [5]. These findings underscore the rapid evolution of centromeres between species and highlight the complex regulatory mechanisms underlying these processes, which remain largely unexplored.

Epigenetic investigations have provided insights into the unique chromatin landscape of centromeres. For example, H3K4me2, which is typically associated with active transcription, exhibits an intriguing enrichment pattern at centromeres in humans and *Drosophila* [16–18], though low levels of this mark are detected in chicken DT40 cells [19]. This duality suggests a context-dependent role for H3K4me2 in centromere biology. Additionally, hypoacetylation of H3 is crucial for centromere function in *Schizosaccharomyces pombe* [20]. In the holocentric *Caenorhabditis elegans*, a positive correlation exists between H3K27me3 and CENP-A domains [21], although the functional significance of this association remains elusive, as reducing H3K27me3 does not affect CENP-A localization [22]. Furthermore, chromatin accessibility at centromeres contributes to its dynamic organization [23]. The three-dimensional (3D) chromatin architecture, including the spatial positioning of centromeres within the nucleus, is vital for proper chromosome segregation [2]. Despite advances in understanding centromere epigenetics, the extent to which these marks are involved in centromere activation or inactivation remains unclear.

**Fig 1:**
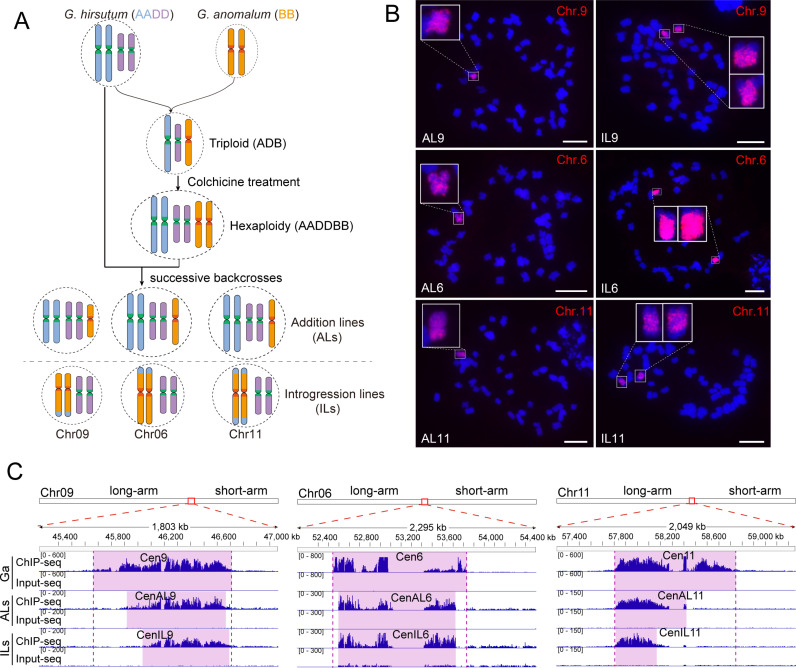
Reduction of Cen6, Cen9 and Cen11 of Ga in the genetic background of Gh. **(A)** Diagram illustrating Gh-Ga monosomic alien addition lines (ALs) and chromosome segment introgression lines (ILs). **(B)** FISH mapping showing Ga chromosome in ALs and ILs. Bars, 10 μm. **(C)** Identification of CENH3 binding region on chromosomes 6, 9 and 11 of Ga. The top track of each panel indicates the position on the corresponding chromosome. The ChIP-seq and Input-seq signal are shown in the blue histogram. Purple shaded areas highlight CENH3-binding domains.

Cotton (*Gossypium*) is a globally significant crop for both natural textile fibers and seed oils. In addition to a natural allopolyploidization event that formed the tetraploid cotton species (AD-genome) about 1–2 million years ago, the availability of interspecific synthetic hybrids, chromosome addition lines (ALs), and chromosome segment introgression lines (ILs) [24,25] offer unique opportunities to investigate centromere dynamics and evolution. Here, we performed CENH3 ChIP-seq to characterize centromeres in *Gossypium hirsutum* (AD-genome), *G. anomalum* (B-genome), their interspecific hexaploid hybrid (ADB-genome) [26], and Ga ALs and ILs in the Gh background (Fig 1A). Aiming to elucidate centromere evolution and regulation accompanying interspecific crossing and subsequent backcrossing, we explored centromere position and composition, the epigenetic landscapes involving histone modifications, and alterations in open chromatin and 3D chromatin conformation. This work enhances our understanding of centromere biology in cotton and contributes to the general principles of centromere evolution in plants, offering a foundation for future research and potential applications in crop improvement.

## Results

### Contraction of *G. anomalum* centromeres in the genetic background of *G. hirsutum*

To investigate centromere variation, we performed CENH3 chromatin immunoprecipitation and sequencing (ChIP-seq) on the B-genome diploid *G. anomalum* (Ga) and on two types of lines containing Ga centromeres in the genetic background of *G. hirsutum* (Gh): the aneuploid or addition line AL9, which contains Ga chromosome 9, and the introgression line IL9, where the majority of Gh chromosome A09 is replaced by Ga chromosome 9 (Fig 1A and 1B and S1 Table). The CENH3-binding domain of Ga chromosome 9 (Cen9) was mapped between 45.67 and 46.71 Mb, covering a 1.04 Mb region (Fig 1C). In AL9, the CENH3-binding domain of Ga*-*derived chromosome 9 (CenAL9) spanned 0.76 Mb (45.91–46.67 Mb), indicating an approximately one-quarter size reduction of the Ga Cen9 in the Gh background. In IL9, the CENH3-enriched regions of chromosome 9 (CenIL9) were only 0.65 Mb (46.04-46.69 Mb), which is about one-third smaller than Cen9 in Ga.

To determine whether this size reduction is a general phenomenon, we conducted CENH3 ChIP-seq on four additional lines, AL6, IL6, AL11, and IL11, which contain Ga-derived centromeres 6 and 11 (Fig 1A and 1B). Despite the presence of gaps existing in the centromere assemblies of Ga chromosome 6 (~296 kb) and 11 (~53 kb), size-reductions were consistently observed in the centromeres of all AL and IL lines compared to Ga (Fig 1C). Notably, CenAL11 was 0.61 Mb, 1.69 times greater than CenIL11 that measured 0.36 Mb, indicating different size dynamics between IL and AL lines. Similar results were observed between CenAL9 and CenIL9 (0.76 Mb vs 0.65 Mb).

Interestingly, the size reductions in centromeres were almost unidirectional in both AL and IL lines. Specifically, the reduced regions of both CenAL9 and CenIL9 were consistently observed on the long-arm side (Fig 1C), while those of CenAL11/CenIL11 and CenAL6/CenIL6 primarily occurred on the short-arm side. Sequence composition analyses revealed no discernible differences between the long-arm and short-arm (S1 Fig), suggesting that this variation may not be attributed to DNA sequence compositions. In addition, the commonly identified centromere regions in ALs and ILs exhibited highly consistent CENH3-binding enrichment profiles with Ga, indicating stable CENH3 binding in Als and ILs despite their size changes (S2 Fig).

We further examined non-TE gene activity within the centromeric regions because the active gene may play a role in centromere formation [15]. In the Cen9, Cen6, and Cen11 regions, there is one gene, one gene, and four genes, respectively. Comparative RNA-seq assays between Ga and these six AL and IL lines revealed that, among these six genes, only *Goano06G1609* (located in the Cen6 region, encoding a ubiquitin protein), exhibited significant down-regulation in both AL6 and IL6 compared to Ga (S3 Fig) (absolute log_2_ (fold change) >1 and adjusted p-value < 0.05). This suggests that centromere contraction has a limited impact on gene expression within the centromeric regions, but the down-regulation of *Goano06G1609* may represent a potential target for further investigation in future studies.

### Increased histone modification levels associated with centromere contraction

To investigate the epigenetics of centromere contraction (i.e., reduction in CENH3 binding regions), we performed ChIP-seq assays with antibodies against ten histone modification marks, including seven corresponding to the active transcriptional state (H3K4me1, H3K4me3, H3K9ac, H3K9me3, H3K36me2, H3K36me3, H3K27ac) and three corresponding to the repressive transcriptional state (H3K4me2, H3K27me2, and H3K27me3). In total, 3,613.4 million ChIP-seq reads were generated using leaves from Ga, AL9, and IL9 (S1 Table), with Pearson correlation coefficients between biological replicates greater than 0.93 (S4 Fig). Modification profiles for active and repressive marks displayed positive and negative correlations with gene expression, respectively (S5–S7 Figs).

**Fig 2:**
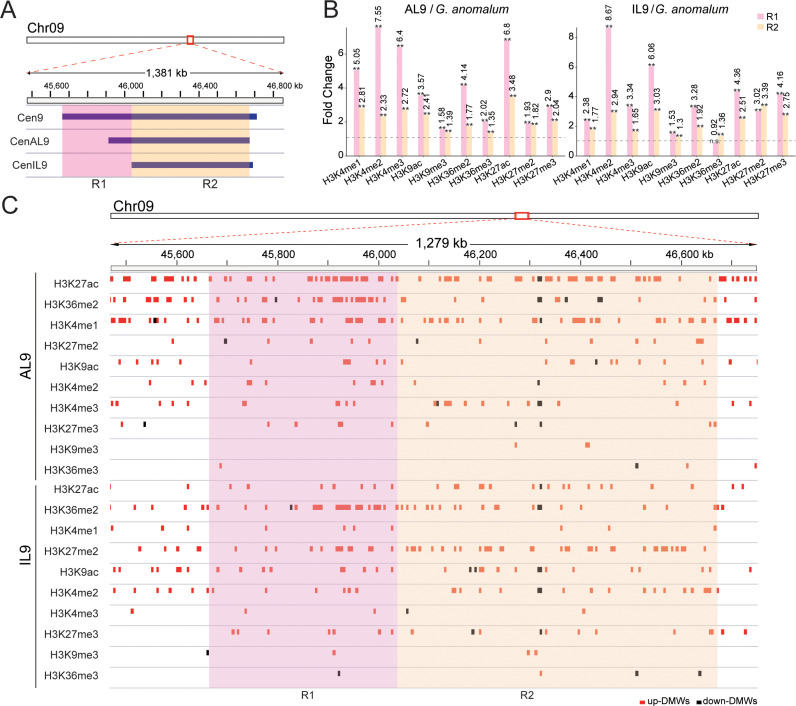
Histone modification dynamics in the Cen9 region. **(A)** Schematic diagram delineating the R1 and R2 regions within Cen9. **(B)** Bar graphs showing the fold changes in histone modification intensities in the R1 and R2 regions for AL9 and IL9 compared to Ga. Normalized histone modification intensities were calculated as the number of ChIP-seq reads within the region divided by the total read count. These normalized intensities were then used to calculate fold changes. Fisher’s exact test was used to analyze statistical significance: **P < 0.01, *P < 0.05, n.s., not significant. **(C)** IGV tracks displaying the distribution of identified DMWs in the R1 and R2 regions for AL9 and IL9 compared to Ga.

Much weaker signals were observed for all ten markers within the centromeres compared to the chromosome arms (S8 Fig), consistent with previous studies [18,27–29]. To detail the dynamic changes in chromatin state within centromeres, we partitioned centromeric regions into variable (R1, 0.37 Mb, where CENH3 binding was lost) and stable regions (R2, 0.64 Mb, where CENH3 binding was conserved) based on the changeability of CENH3 binding in CenAL9 and CenIL9 relative to Cen9 (Fig 2A). We then calculated normalized ChIP-seq signals for R1 and R2 regions in the three lines. Compared to Ga, AL9 and IL9 demonstrated substantial increase in ChIP-seq signals in R1 regions for all ten marks (Fold changes 1.53-8.67), except for H3K36me3 in IL9 (p-value < 0.01, Fisher’s exact test) (Fig 2B). Increased ChIP-seq signals were also observed from R2 regions of both AL9 and IL9 (Fold changes 1.30-3.48) (p-value < 0.01, Fisher’s exact test). Intriguingly, R1 exhibited greater increment in ChIP-seq signals than R2 regions in both AL9 and IL9 (except for H3K36me3 and H3K27me2 in IL9) (Fig 2B). These findings indicate an asymmetric elevation of histone modification in both variable and stable centromeric regions, i.e., variable regions demonstrated higher levels of up-regulated histone modifications.

To quantify dynamic changes of histone modification, we counted ChIP-seq reads within 5kb non-overlapping windows across Ga chromosomes Chr09. Significant changes in histone modification levels were determined by comparing AL9 and IL9 with Ga (fold change > 1.5 and adjusted p-value < 0.05)*.* This analysis revealed 640–5894 differential modification windows (DMWs) for each mark in the Ga-derived Chr09 in AL9 and IL9 lines (S9 Fig). In the centromeric regions, we observed at least ten DMWs for six marks (H3K27ac, H3K36me2, H3K27me2, H3K9ac, H3K4me2, H3K27me3) in both AL9 and IL9 lines, with the majority being up-regulated (150 out of 163 DMWs in AL9 and 172 out of 183 in IL9) (Figs 2C and S10). Notably, we observed a substantial abundance of up-regulated DMWs associated with H3K36me2 in both AL9 and IL9, predominantly clustering in the R1 region (Figs 2C and S11), suggesting a role for H3K36me2 in centromere contraction. It is worth noting that cross-species comparisons and the subjective nature of window size partitioning introduces the potential for false positives in the results presented above. Despite these limitations, all of our results from different analytical strategies revealed a robust association between elevated histone modifications levels and centromere contraction.

### Dynamic changes of histone modification extend to pericentromere in both AL and IL lines

**Fig 3:**
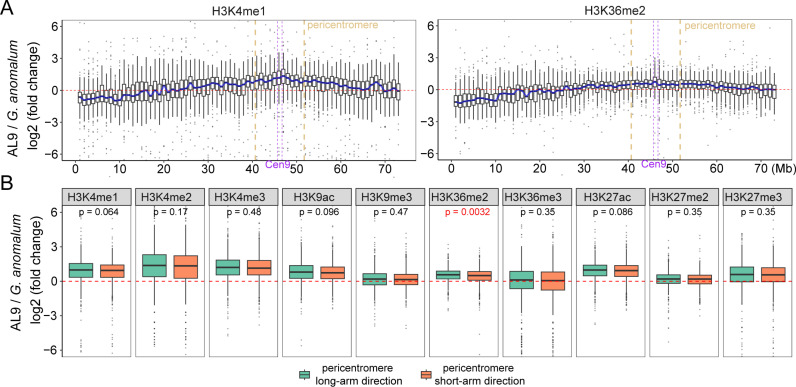
Histone modification dynamics in the pericentromeric regions. **(A)** Box plots showing the dynamics of H3K4me1 and H3K36me2 intensities on chromosome 9 of Ga in the Gh genetic background. Each box represents a 1 Mb region. Light brown dotted lines indicate the 5 Mb pericentromeric regions flanking the centromere. **(B)** Box plots displaying the changes in histone modification intensities within the 5 Mb regions on the long arm and short arm sides of the Cen9. Wilcoxon test was used to determine the significance of the differences between these regions.

We further examined the pericentromeric regions, defined as the 5 Mb regions flanking the centromeres, to investigate whether histone modification changes extended beyond the centromeres themselves. Compared to Ga, elevations in histone modifications were observed in the pericentromeric regions for eight of the ten marks (except for H3K9me3 and H3K36me3) in both AL9 and IL9 (Figs 3A and S12–S14). Notably, the extent of these elevated modification diminished as the distance from the centromeres increased. We noted that the increases in histone modification within the pericentromeric regions displayed an overall symmetric pattern in both long- and short-arm directions for six marks (H3K4me1, H3K4me3, H3K9ac, H3K27ac, H3K27me2, H3K27me3) (Figs 3B and S15). However, H3K36me2 showed a more pronounced increase in the long-arm direction (p < 0.05, Wilcoxon test) (Figs 3B and S15), where centromere contraction occurred. Connecting these findings with the previous section, we observed a substantial abundance of DMWs associated with H3K36me2 in both AL9 and IL9, predominantly clustering in the variable centromeric regions (R1). This suggests that the increase in H3K36me2 levels is not only correlated with centromere contraction but also extends into the pericentromeric regions, implying a broader chromatin remodeling effect. The enrichment of H3K36me2 both within and around the centromeres highlights its significant role in mediating these structural changes. These findings collectively demonstrate that elevation of multiple histone modification levels within the centromere and pericentromere is closely associated with centromere size reduction in AL and IL lines.

Furthermore, we examined the correlation between histone marks and gene expression changes within or around the centromeric regions. In Ga chromosome Chr09, a total of 93 genes were identified within the centromeric or pericentromeric regions, including one gene located at the centromere, 35 genes upstream, and 57 genes downstream (S16 Fig). We evaluated their differential expression in AL9 and IL9 compared to Ga and assessed the presence of DMWs. This analysis revealed that all differentially expressed genes exhibited DMWs within their genomic regions or their 1 kb flanking regions, with most showing downregulated DMWs for both active and repressive histone marks. Interestingly, DMWs were also present in some genes that did not show significant expression changes (S16 Fig). These findings suggest a complex regulatory relationship between histone modifications and transcriptional activity in centromeric and pericentromeric regions.

### Centromere contraction and histone modification dynamics in hexaploid cotton

We analyzed the hexaploid (AADDBB) cotton (Fig 1A) plant to investigate whether centromere constriction occurs after polyploidization. CENH3 ChIP-seq assays (S1 Table) showed that all Ga centromeres underwent size reductions in the hexaploid (S2 Table and S17 Fig). The centromere sizes of Ga chromosomes 6, 9, and 11 were similar to those in either AL or IL lines, indicating that centromere size changes may occur immediately after synthetic polyploidization.

**Fig 4:**
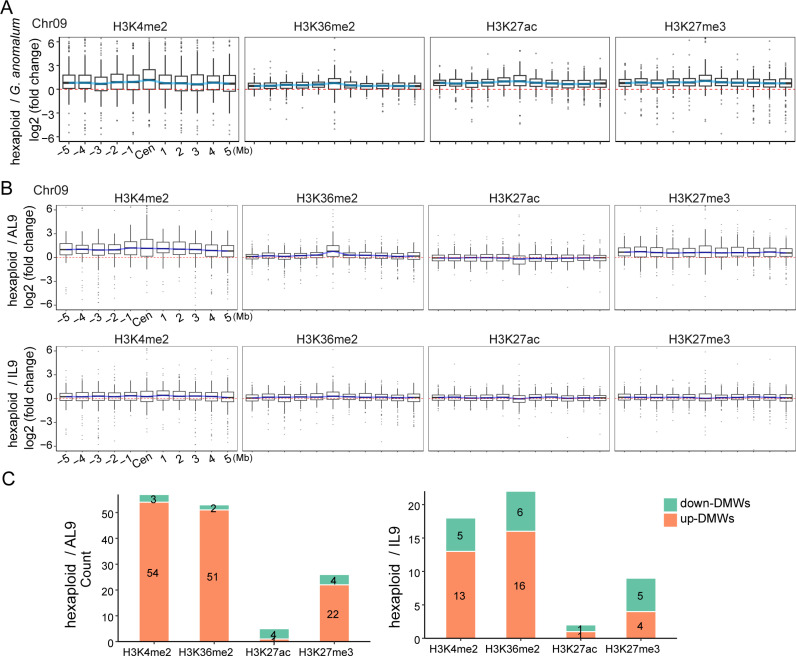
Histone modification dynamics of the Ga centromere 9 in the hexaploid genome. **(A)** Box plots showing the dynamics of histone modification intensities of Ga centromere 9 by comparing the hexaploid to Ga. **(B)** Comparing the histone modification intensities of Ga centromere 9 between hexaploid genome with AL9 or IL9. **(C)** Bar graphs displaying the number of DMWs identified in the centromere region of Ga chromosome 9 within hexaploid cotton by comparing to AL9 (left panel) or IL9 (right panel).

ChIP-seq analysis of histone modifications around Ga centromeres in the hexaploid plant (S1 Table) showed consistently increased signal intensities for all four tested marks, H3K4me2, H3K36me2, H3K27ac, and H3K27me3, compared to Ga (Figs 4A and S18). Elevated signals were also observed in the pericentromeric regions of all Ga chromosomes in the hexaploid (Figs 4A and S18). Furthermore, we identified DMWs associated with these histone modifications. In the centromeric regions, we identified 513 up-regulated DMVs for H3K36me2, 474 for H3K27ac, 706 for H3K27me3, and 78 for H3K4me2 across all 13 centromeres (S19 Fig), underscoring the significant increase in histone modifications accompanying centromere contraction upon hexaploid formation.

Interestingly, H3K36me2 and H3K4me2 levels in CenAL9 and CenIL9 were higher in the hexaploid than in the addition or introgression lines, with 13–54 up-regulated DMWs and fewer than 6 down-regulated DMWs (Fig 4B and 4C). In contrast, fewer than 4 DMWs (up- or down-regulated) were detected for H3K27ac when comparing Cen9 in the hexaploid with CenAL9 and CenIL9. We noted that centromere sizes of Cen9 (0.62 Mb) in the hexaploid are different, to an extent, with that of CenAL9 (0.76 Mb) and CenIL9 (0.65 Mb). Thus, these results may indicate that some histone modifications, such as H3K36me2 and H3K4me2, are sensitive to the centromere size dynamics, and may indicate a role for these epigenetic marks in centromere size regulation.

### A role for chromatin accessibility in centromere contraction?

**Fig 5:**
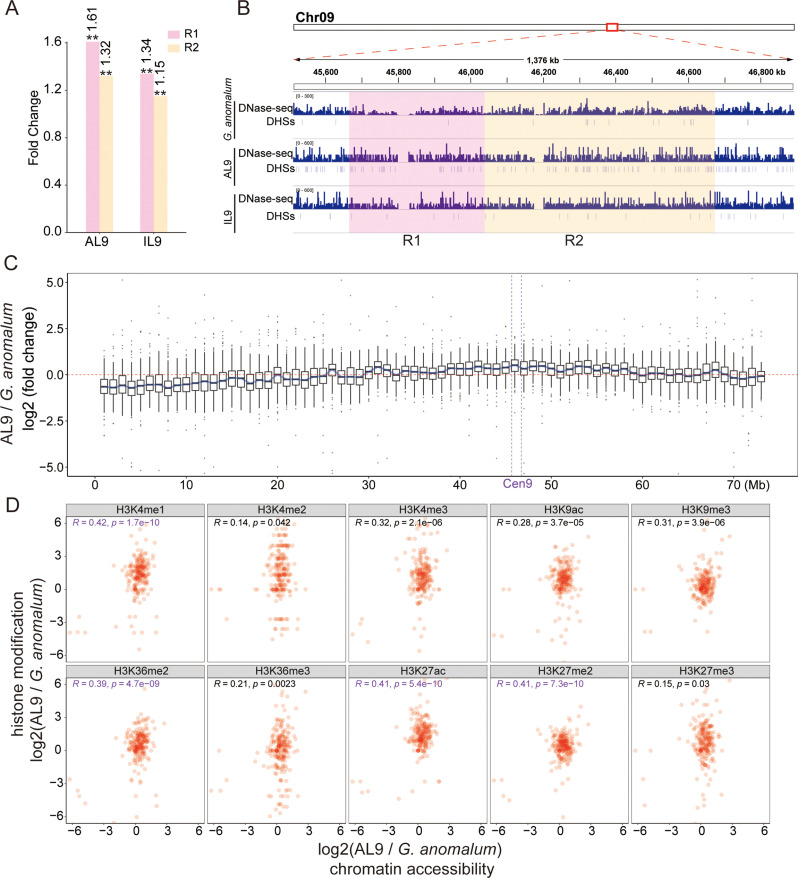
Chromatin accessibility dynamics of Ga chromosome 9 in AL9 and IL9. **(A)** Bar graphs showing the fold changes in chromatin accessibility intensities in the R1 and R2 regions for AL9 and IL9 compared to Ga. Normalized chromatin accessibility intensities were calculated as the number of DNase-seq reads within the region divided by the total read count. These normalized intensities were then used to calculate fold changes. The Fisher’s exact test was used to analyze statistical significance: **P < 0.01, *P < 0.05, n.s., not significant. **(B)** IGV tracks displaying the distribution of identified DHSs in the R1 and R2 regions for Ga, AL9, and IL9. **(C)** Box plots showing the dynamics of chromatin accessibility intensities on Ga chromosome 9 in AL9. Each box represents a 1 Mb region. **(D)** Correlation between changes in chromatin accessibility intensities and histone modification intensities in the Ga centromere 9 in AL9.

We investigated the potential role of chromatin accessibility in promoting the epigenetic and centromere size dynamics observed in our experimental crosses. DNase-seq assays from Ga, AL9, and IL9 (S1 Table) demonstrated reduced chromatin accessibility in centromeres compared to chromosomal arms (S20 and S21 Figs). Notably, both AL9 and IL9 exhibited significantly increased DNase-seq signals in both R1 and R2 regions compared to Ga, with fold changes of 1.61 and 1.34 in R1 and 1.32 and 1.15 in R2, respectively, with a more pronounced increase in R1 (p-value < 0.01, Fisher’s exact test) (Fig 5A). This increase in DNase I-hypersensitive sites (DHSs) in AL9 and IL9 supports the interpretation of increased chromatin accessibility in these lines (Fig 5B). Additionally, increased accessibility was also found in pericentromeric regions of AL9 and IL9, with the signal gradually diminishing towards the distal chromosome ends (Figs 5C and S22). The accessibility increments showed symmetric patterns between long- and short-arm directions (S23 Fig), resembling the histone modification patterns.

Previous studies have established a link between chromatin accessibility and histone modifications [30–32]. We sought to elucidate whether changes in chromatin accessibility at centromeres correlate with alterations in specific histone modifications. To address this, we employed a similar strategy as in histone modification analysis, partitioning Chr09 into 5kb windows and calculating the intensity of chromatin accessibility within each window. Pearson correlation analysis between chromatin accessibility changes and histone modification changes in the Cen9 region revealed a significant positive correlation (R = 0.14-0.67, p < 0.05), particularly for H3K4me1, H3K36me2, H3K27ac, and H3K27me2 in both AL9 and IL9 (Figs 5D and S24).

### Non-B-form DNA in centromere contraction

**Fig 6:**
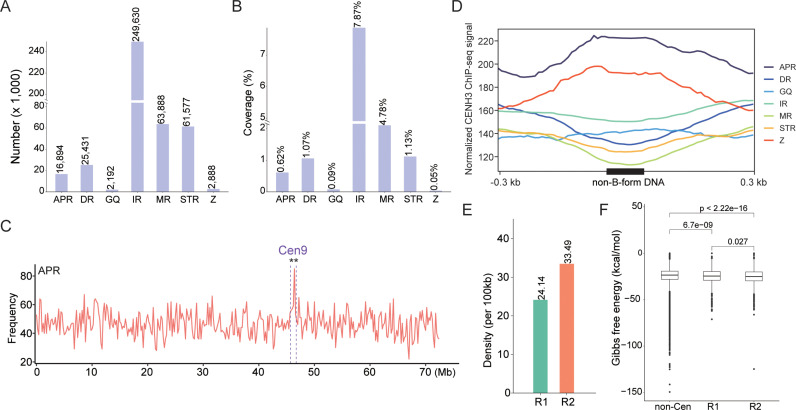
Identification of Non-B-form DNA on Ga chromosome 9. **(A)** The number of Non-B-form DNA on Ga chromosome 9. A-phased DNA Repeat (APR), Direct Repeat (DR), G-quadruplex (GQ), Inverted Repeats (IR), Mirror Repeat (MR), Short Tandem Repeat (STR), and Z-DNA motifs **(Z)**. **(B)** Coverage of Non-B-form DNA on Ga chromosome 9. **(C)** Distribution density of Non-B-form DNA across Ga chromosome 9. Ga chromosome 9 was divided into non-overlapping 200 kb windows, and the density of Non-B-form DNA in each window was plotted as a line graph. The centromeric region is marked with purple dashed line. A permutation test (n = 1000) was used to determine if the density of Non-B-form DNA in the centromeric region was higher than in non-centromeric regions: **P < 0.01, *P < 0.05, n.s., not significant. **(D)** Distribution of CENH3 ChIP-seq read signals on non-B-form DNA. **(E)** Bar graph showing the density (numbers per 100 kb) of APR in the R1 and R2 regions. **(F)** Box plots displaying the Gibbs free energy in the R1, R2, and non-centromeric regions. The Wilcoxon test was used to determine the significance of differences between these regions.

Non-B-form DNA plays critical roles in regulating centromere stability and influencing CENH3 loading [13,33]. To investigate the potential association between centromere contraction and non-B-form DNA, we employed the non-B-form DNA motif search tool (nBMST) to predict seven different non-B-form DNA motifs. This analysis revealed 2,192–249,630 non-B-form DNA motifs within Ga Chr09, comprising 0.05%-7.87% of the genomic DNA content (Fig 6A and 6B). Examination of the distribution patterns of non-B-form DNA motifs along Chr09 revealed that A-phased DNA Repeats (APRs) exhibited significantly elevated frequency in the centromeric region compared to non-centromeric regions (p-value < 0.01, permutation test) (Fig 6C). APRs are non-B-form DNA tracts of repeats of three to nine adenines or thymines (A-tracts) separated by at least a 4 bp spacer, facilitating bent double-helix structures [34]. Notably, APRs also showed high CENH3 ChIP-seq signal (Fig 6D). In contrast, the remaining six types of non-B-form DNA displayed either decrease or stable density in centromere compared to non-centromeric regions (S25 Fig). These observations suggest APRs may play a role in centromere function.

We also compared the density of APRs between R1 and R2 regions, revealing that the density of APRs in R2 regions was 1.39 times higher than in R1 regions (Fig 6E). We computed the Gibbs free energy in R1 and R2 regions, where lower free energies indicate a more stable DNA secondary structure. We found that R2 regions exhibited lower Gibbs free energy (average -24.8 kcal/mol in R1 region, -25.1 kcal/mol in R2 region) (p < 0.05, Wilcoxon test) (Fig 6F), suggesting increased thermodynamic stability of DNA secondary structures in the R2 regions. Similar results were observed in Ga Chr11 (S26 Fig), where APR content was abundant within centromeric regions, with stable centromeric regions in AL11 and IL11 harboring a higher density of APRs and lower Gibbs free energy compared to variable regions. Additionally, despite the presence of a noticeable gap (~296 kb) in the centromere of Ga Chr06, APR-form DNA was still significantly enriched at this centromere (p-value < 0.01, permutation test) (S27 Fig). Together, these findings suggest a correlation between APR content and CENH3 binding stability, where centromeric variable regions with fewer APRs may be more prone to the loss of CENH3 binding compared to stable regions.

### 3D chromatin reorganization accompanied by centromere contraction

**Fig 7:**
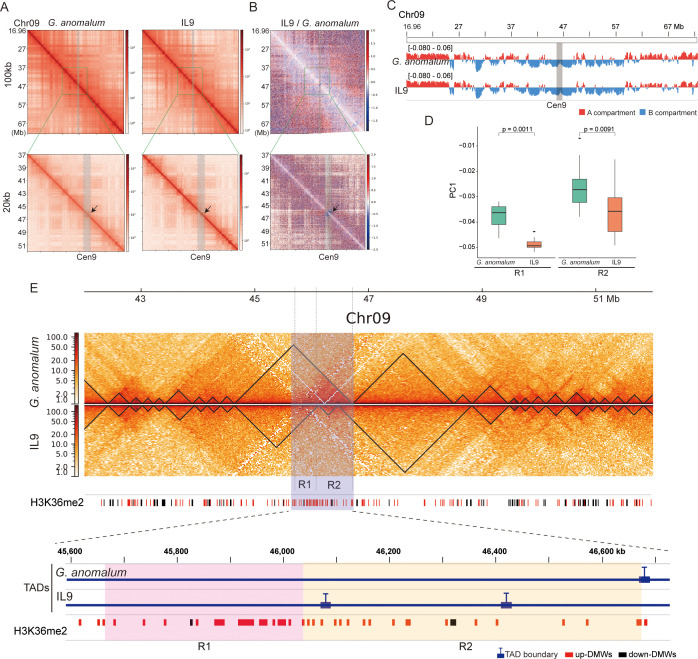
Comparison of chromatin interaction profiles between Ga and IL9. **(A)** Multiresolution Hi-C contact heatmaps for Ga and IL9. Normalized Hi-C matrices were constructed at resolutions of 100 kb and 20 kb. The arrows indicate the Cen9 region. **(B)** Hi-C heatmaps of relative contact differences between Ga and IL9. The relative difference is calculated as the log_2_ ratios of the Hi-C matrices of the IL9 and Ga samples. In the heatmap, Hi-C interactions that become stronger in IL9 compared to Ga are in white to red, whereas Hi-C interactions that become weaker in IL9 compared to Ga are in white to blue. Hi-C interactions that do not change between IL9 and Ga are in white. **(C)** Distribution of A and B compartments on Ga chromosome 9. **(D)** Comparison of PC1 values obtained from the Hi-C data for Ga and IL9. The Wilcoxon test was used to determine the significance of differences. **(E)** Identification of TADs on Ga chromosome 9. TADs identified as regions of high chromatin interactions are outlined by black triangles. The bottom track shows the distribution of H3K36me2 DMWs.

To explore the role of 3D chromatin architecture in centromere contraction, we conducted Hi-C experiments using leaves from Ga and IL9, generating a total of 2,712.1 million sequencing read pairs (344× for the Ga genome and 173× for the IL9 genome) (S1 Table). For IL9, only the reads corresponding to the Ga-derived chromosome segment (Chr09:16.96-72.55 Mb) were extracted and used in further analysis. Hi-C matrix analysis at different resolutions revealed hierarchical, higher-order chromatin structures (Fig 7A).

We first investigated the global Hi-C contact maps for the Chr09:16.96-72.55 Mb segment in Ga and IL9, noting significant changes in interaction frequency, particularly within the centromeric region, where interactions were diminished (Fig 7B). Chromatin was partitioned into A and B compartments, representing putatively active and inactive chromatin regions, respectively. We further explored the dynamics of compartments at the centromere, using PC1 values from principal component analysis (PCA) of the Hi-C contact matrices. Positive and negative PC1 values indicate A and B compartments, respectively. The Cen9 region was categorized as a B compartment in both Ga and IL9 (Fig 7C), consistent with its heterochromatic nature and centromeric inactivity. However, PC1 values for the Cen9 region in IL9 were significantly reduced compared to Ga (p < 0.01, Wilcoxon test), particularly in the R2 regions (stable centromeric regions) (Fig 7D). Specifically, Ga exhibited mean PC1 values of -0.0378 in R1 (variable centromeric regions) and -0.0263 in R2, while IL9 displayed mean PC1 values of -0.0486 in R1 and -0.0357 in R2, reflecting a 1.29-fold reduction in R1 and a 1.36-fold reduction in R2. These data indicate that chromatin organization in the IL9 centromere is altered compared to its native state in Ga.

We also studied topologically associating domains (TADs), which are fundamental structural units of the 3D genome [35,36]. We identified 162 TADs in Ga and 163 TADs in IL9. Comparative analysis of the Cen9 region (Figs 7E and S28) revealed that in Ga, Cen9 resided within a single TAD, whereas in IL9, two new TAD boundaries emerged within Cen9, separating R1 and R2 into distinct TADs. Notably, one of these new TAD boundaries emerged between R1 and R2, suggesting a potential functional significance. However, given the complexities of TAD identification, further experimental validation is necessary to confirm the accuracy of these findings. Interestingly, this new TAD boundary overlapped with upregulated H3K36me2 DMWs (Fig 7E). Although we cannot establish a causal relationship between H3K36me2 upregulation and the formation of TAD boundaries, previous studies have demonstrated a relationship between H3K36me2 and TAD boundary dynamics [37], suggesting that these changes may be associated with increased levels of H3K36me2. Together, these findings suggest that the alien Ga Cen9 in the Gh background stimulates rearrangement of chromatin interactions.

## Discussion

Centromeres are indispensable for faithful segregation of chromosomes during cell division, as they provide the structural basis for kinetochore assembly [38,39]. They are also extraordinarily diverse and variable among species, yet the processes governing centromere divergence and evolution are poorly understood, especially in the context of genomic perturbations such as hybridization or polyploidy. Here we tracked the dynamics of alien centromeres from the diploid *Gossypium anomalum* (Ga) in the genomic background of the allopolyploid cotton species *G. hirsutum* (Gh) to explore the consequences of interspecific genomic merger on centromere size, epigenetic state, and chromatin architectural features that might affect function, including accessibility and 3D organization.

Our observation of the contraction of Ga centromeres when introduced into the Gh genetic background underscores the dynamic nature of centromeres and their ability to rapidly change in new genomic environments (Figs 1C and 4A). This finding, which is consistent with emerging evidence that centromeres undergo dynamic changes in response to genetic perturbations [5,15,40–42], highlights the plasticity of centromeric chromatin. In maize, backcrossing known centromeres into lines with larger genomes resulted in consistent increases in functional centromere sizes across multiple centromeres [43]. In particular, maize centromeres (average size 2.2 Mb) expanded dramatically when transferred into the oat genome (average centromere size 6.9 Mb) [13–15], with these expanded regions being epigenetically and transcriptionally activated [44]. These findings suggests that centromere size is regulated and foreign centromeres will undergo adaptive adjustment to be uniform within a new genomic context. In our study, we observed the contraction of Ga centromeres when introduced into the Gh background. It is consistent the centromere size adaption as all Ga centromeres studied are greater than Gh counterparts. Thus, our findings enhance the centromere size adaptive adjustment when hybridization between two species with distinct centromere sizes. We observed that the majority of Gh centromeres maintained remarkable size stability across hexaploid, AL, and IL cottons. However, a subset of centromeres exhibited distinct size variations (S29 Fig).

Of likely consequence is our observation of notable increases in specific histone modifications, such as H3K36me2, H3K4me2, H3K9ac, and H3K27ac, within the contracted centromeres (Fig 2). These modifications, typically associated with active transcription and euchromatin [30,45–47], increase the likelihood that instant epigenetic alterations accompanying genomic merger have functional consequences on gene expression and hence biological function [48–50]. Notably, H3K27me3, typically associated with gene repression and heterochromatin, is present in the contracted centromeres. In *C. elegans*, a positive correlation or co-localization has been observed between H3K27me3 and CENP-A (known as CENH3 in plants) domains [21,51]. Centromeric H3K27me3 also naturally occurs in human cell lines [52,53]. In mouse, mitotic chromosomes from cells lacking H3K9me3 were found to be significantly more compact and decorated with H3K27me3 [54]. Therefore, the deposition of H3K27me3 in the contracted centromeres may be part of a broader chromatin remodeling mechanism essential for proper centromere function.

The coexistence of histone marks with opposing functions, such as H3K36me2 and H3K27me3, in centromeric and pericentromeric regions is intriguing (Figs 2 and 3). This suggests the presence of a hybrid chromatin state that integrates euchromatic and heterochromatic features, which may be essential for centromere functionality. While early studies associated centromeres primarily with heterochromatin, more recent research across diverse organisms, including humans, *Drosophila*, and *Arabidopsis*, has demonstrated that centromeres exhibit a unique combination of euchromatic and heterochromatic features, suggesting that this hybrid chromatin state is conserved [18,29,55,56]. In fact, euchromatic marks in the centromere may be essential for its identity and function [55]. Studies on human artificial chromosomes formation and maintenance have shown that artificially increasing heterochromatin at the alpha-satellite array impairs CENP-A deposition and centromere function, whereas the presence of H3K4me2 and elevated H3K9 acetylation promote the maintenance of CENP-A [55,57–59]. Notably, the loss of euchromatic histone modifications, such as H3K4me2 or H3K36me2, occurs during centromere inactivation [60]. Together, our data add to recent studies that have demonstrated the presence of euchromatic features within centromeric chromatin [29,61,62], emphasizing the importance of this hybrid chromatin state in maintaining centromere integrity and function. It is possible that the histone modifications observed serve as signals for chromatin remodeling complexes. These complexes may facilitate the reshaping of centromeric architecture, promoting greater compactness while retaining essential features necessary for kinetochore formation.

The observed differential changes in histone modifications between variable (R1) and stable (R2) centromeric regions underscore the notion that epigenetic mechanisms play a pivotal role in maintaining centromere composition and functionality (Fig 2) [63,64]. The identification of differential modification windows (DMWs) within these regions suggests that targeted epigenetic reprogramming is a fundamental feature of the epigenetic landscape during centromere contraction. These localized changes in histone modification patterns may serve as focal points of epigenetic activity, orchestrating specific modifications in response to genetic perturbations, and which may support centromere function. Given the role of histone modifications in chromatin organization [65–67], the presence of DMWs is believed to involved in various processes, including the formation of higher-order chromatin structures essential for centromere stability. As observed, the contraction of centromeres was associated with the reorganization of TADs within the centromeric region, resulting in the emergence of new TAD boundaries (Fig 7E). While we observe the overlap between new TAD boundaries and H3K36me2-associated DMWs in contracted centromeric regions, a direct causal relationship between them cannot be inferred from this alone. Recent studies have shown that certain histone marks, including H3K36me2, are enriched at TAD boundaries [68–70], and that the expansion of H3K36me2 domains can drive changes in 3D chromatin organization, including alterations in A/B compartmentalization and TAD structure [37]. Thus, the observed overlap in our study may indeed be functionally significant and suggests a potential role for H3K36me2 in centromeric chromatin architecture. However, to establish a definitive causal link, further functional perturbations of H3K36me2 will be necessary.

Our study also sheds light on the broader significance of histone modifications in shaping overall chromatin architecture. Specifically, the expansion of heightened histone modification levels, such as H3K36me2, H3K4me1, and H3K27me2, into pericentromeric regions observed in our study is notable (Figs 3, S12 and S13). It is well-known that histone modifications play a role in protecting genomic integrity. For instance, H3K36me2 has been shown to recruit and stabilize components of the DNA repair machinery, correlating with DNA repair efficiency [71]. Similarly, H3K4me1 is linked to genome stability, DNA repair and lower mutation rates [72]. H3K27me2 is involved in the silencing of euchromatic transposons to maintain genome stability [73]. Thus, the elevated levels of these histone modifications in pericentromeric regions may promote structural integrity and enhance chromosomal stability by creating a more robust chromatin environment that can better withstand genetic perturbations. This mechanism may support the essential functions of centromeres, even as they undergo size and structural changes, which is particularly important in contexts such as polyploidy and hybridization where genomic perturbation is integral to these organismal level processes.

Interspecific hybridization or polyploidization can induce genomic structural variations, including alterations at the centromere, and the lack of an available hexaploid cotton genome presents a challenge in studying centromere dynamics in this context. To address this, we constructed a combined reference genome using the parental Ga and Gh genomes. While this approach may not fully capture all hexaploid-specific centromeric dynamics, it represents a commonly adopted approach for assessing genome changes following interspecific hybridization or polyploidization [30,74–76]. Using this reference, we observed that centromere size patterns and histone modifications in hexaploid cotton were comparable to those in ALs and ILs (Fig 4), suggesting that these epigenetic changes are triggered early during polyploid formation. Polyploidization has been shown to induce extensive epigenetic remodeling in numerous plant groups [77–80]. Our results highlight the epigenetic breadth that this remodeling involves, entailing the upregulation of at least four histone marks at the centromere and pericentromeric regions, including H3K36me2, H3K4me2, H3K27ac, and H3K27me3. Furthermore, H3K36me2 and H3K4me2 are associated with the establishment of an open chromatin state, which can promote centromere assembly [18,58,81]. H3K27ac is linked to chromatin compaction [82]. H3K27me3, typically associated with gene repression, is compatible with centromere stability and mitotic chromosome segregation [52]. The coordinated presence of these marks may ensure that centromeres and other essential genomic regions are properly compacted and functionally stabilized, thus supporting accurate chromosomal segregation during cell division. Notably, the histone marks we examined likely interact with other modifications and epigenetic factors, which together are involved in centromere contraction. For example, several centromere-specific histone modifications, such as H3T3ph, H2AT133ph, and H3S10ph, have been reported to play critical roles in maintaining the structural integrity and functional regulation of centromeres. H3T3ph is involved in the recruitment of key proteins required for centromere assembly as well as chromosome alignment and segregation during cell division [83,84]. H2AT133ph occurs in the CENH3 nucleosome and is crucial for spindle checkpoint control and localization of the centromere cohesion protector Shugoshin [2,85]. H3S10ph has been suggested to play a role in the cohesion of sister chromatids in plants [83]. Future research focusing on elucidating the molecular mechanisms through which these modifications, either independently or in concert, influence centromere architecture and function are likely to be fruitful.

## Materials and methods

### Plant materials

All cotton materials, including the allopolyploid *G. hirsutum* (Gh, AADD genome), the diploid *G. anomalum* (Ga, BB genome), and their tri-genomic interspecific hexaploid (AADDBB genome), and subsequent development of ALs and ILs (including AL6, IL6, AL9, IL9, AL11, and IL11), were grown under controlled greenhouse conditions (13 h/11 h light/dark, 28°C/26°C light/dark, 60% humidity, and 270 µmol/m^2^/s light intensity). Young leaves were collected and immediately frozen in liquid nitrogen for subsequent experiments. To ensure that leaves from different cotton lines were at the same developmental stage, the third and fourth true leaves were harvested (when the fifth true leaf emerged) for ChIP-seq, DNase-seq, RNA-seq and Hi-C library constructions.

### ChIP-seq and data analysis

ChIP-seq assays were conducted following a published protocol [6]. Ten commercial antibodies targeting H3K4me1 (Abcam, Cat. #ab8895), H3K4me2 (Abcam, Cat. #ab7766), H3K4me3 (Sigma, Cat. #07-473), H3K9ac (Sigma, Cat. #06-942), H3K9me3 (Sigma, Cat. #07-442), H3K36me2 (Abcam, Cat. #ab9049), H3K36me3 (Abcam, Cat. #ab9050), H3K27ac (Sigma, Cat. #07-360), H3K27me2 (Sigma, Cat. #07-452), and H3K27me3 (Sigma, Cat. #07-449) were used for ChIP. ChIP experiments included two biological replicates. Additionally, an antibody specific to the cotton CENH3 protein was used [6]. Input DNA served as the control. ChIP and Input DNAs were ligated with Illumina sequencing adaptors, size-selected, PCR-amplified, and sequenced on the Illumina NovaSeq platform to produce 150-bp paired-end reads.

Raw reads generated from ChIP-seq were quality filtered and trimmed using trim_galore v.0.6.7(https://www.bioinformatics.babraham.ac.uk/projects/trim_galore/). Cleaned reads were mapped to their respective genome using Bowtie2 v.2.2.5 [86] with default parameters. Reference genome sequence and annotation files for Ga (B1_JAAS) and Gh (ZJU-v2.1) were downloaded from CottonGen (https://www.cottongen.org/). The hexaploid cotton genome was created by combining the genome sequences of Ga and Gh. The ALs and ILs genome were generated by combining the corresponding Ga chromosomes and Gh genome. Mapped reads were filtered using SAMtools v.1.9 [87] to retain only correctly read pairs with a mapping quality score of 10 or higher. CENH3 peaks were identified using MACS2 v.2.1.4 [88] with ChIP-seq reads alignment set as the treatment and input reads alignment as control. Peaks with a fold change (ChIP/Input) higher than 4 were retained, and the chromosomal regions corresponding to the peak enrichment were considered the functional centromere regions.

To identify differential modification windows (DMWs), The Ga chromosome Chr09 was divided into 5kb non-overlapping windows using the makewindows function in BEDTools v.2.26.0 [89]. ChIP-seq read counts for each window were determined using the multicov function in BEDTools. These counts were then processed using edgeR [90] to perform differential comparisons: (1) between AL9 and Ga, and (2) between IL9 and Ga. DMWs were defined as windows with a fold change greater than 1.5 and an adjusted p-value less than 0.05.

### DNase-seq and data analysis

DNase-seq library construction was performed according to our previously published protocol [91]. Briefly, approximately 1 g of finely ground powder was suspended in an equal volume of pre-chilled nuclear isolation buffer (NIB; 10 mM Tris-HCl, 80 mM KCl,10 mM EDTA, 1 mM spermidine, 1 mM spermine, 0.15% mercaptoethanol, 0.5 M sucrose, pH 9.5) for nuclei isolation. Extracted nuclei were resuspended in nuclear digestion buffer (NDB; 10 mM Tris-HCl, 10 mM NaCl, 3 mM MgCl2, pH 7.5) and digested with gradient concentrations of DNase I for 10 min at 37°C. DNA fragments of with size <250 bp from the optical DNase I treatment were isolated for library construction using NEBNext Ultra DNA Library Prep Kit (NEB, Cat. #E7370). The libraries were developed from two biological replicates for each cotton line and sequenced on the Illumina NovaSeq platform with a 150-bp pair-end model.

Data processing, including read cleaning and mapping steps, was performed as described above for ChIP-seq. Reads mapping to chloroplast or mitochondrial genomes were removed prior to further analysis. DHSs were identified using MACS2. We divided Ga chromosomes into 5 kb non-overlapping windows, quantified the number of DNase-seq reads in each window, and used edgeR to calculate the fold change in chromatin accessibility between different cotton lines within each window.

### RNA-seq and data analysis

Total RNA of leaves from two replicates was extracted using the Omega Plant RNA kit (Omega Bio-tek, Cat. #R6827-01). RNA-seq libraries were prepared using the Illumina TruSeq RNA Kit (NEB, Cat. #E7530) and were sequenced on an Illumina NovaSeq system to generate 150-bp paired-end reads. Clean reads were mapped to the respective genome using Tophat2 v.2.1.1 [92] with default settings. Cufflinks v.2.2.1 [93] was used to calculate gene expression levels (FPKM).

### Hi-C and data analysis

Hi-C library construction was conducted at Frasergen Co., Ltd (Wuhan, China). Approximately 2 g of leaf tissue from two replicates was ground to a powder in liquid nitrogen for the Hi-C experiment. The experimental procedure, including DNA cross-linking, chromatin digestion using the *Mbo*I restriction enzyme, biotin labeling of DNA ends, in situ ligation of proximal ends, reversal of crosslinking, and DNA purification, followed a previously published protocol [94]. The Hi-C library was sequenced using 150 bp paired-end reads on an Illumina NovaSeq platform.

Cleaned read pairs were individually aligned to the respective genome using the BWA-MEM algorithm implemented in BWA v.0.7.17-r1188 software (http://bio-bwa.sourceforge.net) with parameters “-A1 -B4 -E50 -L0”. To compare Ga and IL9, only reads that mapped to the Ga-derived chromosome segment (Chr09:16.96-72.55 Mb) were extracted and used in subsequent analysis. The hicBuildMatrix, hicNormalize, hicCorrectMatrix and hicCompareMatrices from HiCExplorer v.3.7.2 [95] were used to construct 20 kb, 50 kb, and 100 kb Hi-C matrices, normalized for sequencing depth, perform Knight-Ruiz (KR) correction, and generate log_2_ ratios of interaction frequency matrices between IL9 and Ga samples, respectively. Hi-C contact maps were visualized using hicPlotMatrix from HiCExplorer and pyGenomeTracks v.3.7 [96]. To identify chromatin compartments, hicPCA from HiCExplorer was employed with a bin resolution of 50 kb. TADs and their boundaries were identified using hicFindTADs from HiCExplorer at 20 kb resolution with parameters “--correctForMultipleTesting fdr --thresholdComparisons 0.01 --delta 0.01”.

### Identification of non-B-form DNA

Non-B-form DNA motifs in the Ga genomic sequence were identified using nBMST [97]. RNAfold v.2.4.13 from the ViennaRNA package [98] was employed to calculate the folding free energies of non-B-DNA structures. A more negative folding free energy indicates greater stability of the non-B-DNA structure. This analysis utilized a 300 nt sliding window with a 150 nt step size across chromosomes and employed the parameter file dna_mathews2004.par, specifically designed for folding single-stranded DNA sequences.

### Data visualization

For data visualization, BAM files were converted into the bigwig format using the bamCoverage function from deepTools v.3.1.3 [99], employing a bin size of 10 bp and performing RPKM normalization. Average plots illustrating ChIP-seq read signals across gene regions were generated using the computeMatrix and plotProfile functions within the deepTools package. Genome browser images were made using the Integrative Genomics Viewer (IGV) v.2.3.92 with bigwig files processed as described above.

### FISH assay

FISH experiments were conducted following a previous study using Ga chromosome-specific panting probes [100]. Briefly, mitotic chromosome spreads were prepared from root tips, and slides containing well-spread metaphase chromosomes were selected for FISH analysis. Following hybridization, chromosomes were counterstained with 4’, 6-diamidino-2-phenylindole (Vector Laboratories, USA), and the fluorescent signal was detected under an Olympus BX63 fluorescence microscope. Chromosome morphology and FISH signals were captured using cellSens Dimension 1.9 software with an Olympus DP80 CCD camera.

## Supporting information

S1 Fig Sequence composition of Ga chromosomes 6, 9, and 11. Ga chromosomes were divided into non-overlapping 1 Mb windows. The sequence composition within each window was analyzed, including the proportion of repeat sequences (left), gene density (middle), and GC content (right). The red dashed lines indicate the centromeric regions, and the gray shaded areas represent regions that are further magnified using 100 kb windows.
(TIF)

S2 Fig Identification of CENH3 subdomains on Ga chromosomes 6, 9, and 11 in Ga, ALs, and ILs. The top track of each panel indicates the position on the corresponding chromosome. The ChIP-seq signals are shown in blue histograms. The blue bars below the ChIP-seq tracks represent the CENH3 subdomains.
(TIF)

S3 Fig Expression levels of centromeric genes on Ga chromosomes 6, 9, and 11, and their expression dynamics in ALs and ILs. The numbers in the heatmap represent the log_2_ fold changes in gene expression. Asterisks indicate statistical significance (*p < 0.05, **p < 0.01).
(TIF)

S4 Fig Correlation of ChIP-seq data between replicates. The genome was divided into non-overlapping 500 bp windows, and the numbers of ChIP-seq reads in each window were calculated. The correlation heatmap was generated with deepTools software. Colors represent the correlation coefficients, where red indicates high similarity, and blue indicates low similarity. Hierarchical clustering is shown to the left of the heatmap.
(TIF)

S5 Fig Distribution of histone modifications surrounding genes in Ga. Genes were grouped based on expression levels. Expressed genes with FPKM values greater than 0 were equally divided into 3 groups, with high, medium and low expression levels.
(TIF)

S6 Fig Distribution of histone modifications surrounding genes in AL9. Genes were grouped based on expression levels. Expressed genes with FPKM values greater than 0 were equally divided into 3 groups, with high, medium and low expression levels.
(TIF)

S7 Fig Distribution of histone modifications surrounding genes in IL9. Genes were grouped based on expression levels. Expressed genes with FPKM values greater than 0 were equally divided into 3 groups, with high, medium and low expression levels.
(TIF)

S8 Fig Distribution of ChIP-seq signals on Ga chromosome 9. The IGV browser shows ChIP-seq signal tracks for ten histone marks in Ga, AL9, and IL9. Gray shaded areas indicate the Cen9 region.
(TIF)

S9 Fig Number of identified DMWs on Ga chromosome 9. Ga chromosome 9 was divided into non-overlapping 5 kb windows. Differentially modified windows (DMWs) on Ga chromosome 9 in the Gh background were identified based on a fold change > 1.5 and an adjusted p-value < 0.05. Up-DMWs represent DMWs upregulated in the Gh background, while down-DMWs represent those downregulated in the Gh background.
(TIF)

S10 Fig Number of identified DMWs in the Cen9 region. Bar graph showing the number of differentially modified windows (DMWs) identified in the Cen9 region. Up-DMWs represent DMWs upregulated in the Gh background, while down-DMWs represent those downregulated in the Gh background.
(TIF)

S11 Fig Density of up-regulated DMWs in the R1 and R2 regions of Cen9. The density of up-regulated DMWs in the R1 and R2 regions was calculated as the number of up-regulated DMWs divided by the length of the respective region.
(TIF)

S12 Fig Comparison of histone modification intensities on Ga chromosome 9 between Ga and AL9. Each box represents a 1 Mb region. The pericentromere refers to the 5 Mb regions flanking the centromere.
(TIF)

S13 Fig Comparison of histone modification intensities on Ga chromosome 9 between Ga and IL9. Each box represents a 1 Mb region. The pericentromere refers to the 5 Mb regions flanking the centromere.
(TIF)

S14 Fig Curve plots showing the variation in histone modification intensities on Ga chromosome 9 between AL9 and Ga (A), and between IL9 and Ga (B). Ga chromosome 9 was divided into non-overlapping 1 Mb windows, and the number of ChIP-seq reads in each window was counted and normalized by sequencing depth, representing the intensity of histone modification signals. The centromeric regions are indicated by the blue bars in each plot.
(TIF)

S15 Fig Box plots displaying the changes in histone modification intensities between Ga and IL9 within the 5 Mb regions on the long arm and short arm sides of the Cen9. The Wilcoxon test was used to determine the significance of the differences between these regions.
(TIF)

S16 Fig Differential gene expression and histone modification patterns in the centromeric and pericentromeric regions. The centromeric region of Ga chromosome Chr09 contains a gene, *Goano09G2439*. The 5 Mb upstream region of the centromere harbors 35 genes, while the 5 Mb downstream region contains 57 genes. The gene row shows colored dots representing differential gene expression in AL9 and IL9 compared to Ga (fold change > 1.5, adjusted p-value < 0.05). In the histone modification row, colored boxes indicate the presence of differential modification windows (DMWs) within the gene regions or their 1 kb flanking regions.
(TIF)

S17 Fig IGV snapshots showing the centromeres and CENH3 binding subdomains on each chromosome of Ga. The CENH3 ChIP-seq track displays the alignment of ChIP-seq reads, with bars in the centromere track indicating centromeric regions. The CENH3 binding subdomains track shows peaks identified by the MACS2 software, representing the CENH3 binding subdomains.
(TIF)

S18 Fig Box plots showing the dynamics of histone modification intensities in the centromere and pericentromere regions of Ga chromosome within hexaploid cotton. Each box represents a 1 Mb region. Boxplots display lower and upper extremes, lower and upper quartiles, and medians.
(TIF)

S19 Fig Number of up-regulated DMWs identified in the centromeric regions of each Ga chromosome when comparing the hexaploid with Ga. The x-axis labels Cen1-Cen13 refer to the centromeres of Ga chromosomes 1–13, and the y-axis represents the number of up-regulated DMWs.
(TIF)

S20 Fig Correlation of DNase-seq data between replicates. DNase-seq reads were quantified for each 1kb bin along chromosomes, and the Pearson correlation coefficient was determined between two biological replicates.
(TIF)

S21 Fig Distribution of DNase-seq signals on Ga chromosome 9. The IGV browser shows DNase-seq signal tracks in Ga, AL9, and IL9. Gray shaded areas indicate the Cen9 region.
(TIF)

S22 Fig Box plots showing the dynamics of chromatin accessibility intensities on chromosome 9 of Ga in IL9. Each box represents a 1 Mb region. Boxplots display lower and upper extremes, lower and upper quartiles, and medians.
(TIF)

S23 Fig Box plots displaying the changes in chromatin accessibility intensities between Ga and AL9/IL9 within the 5 Mb regions on the long arm and short arm sides of the Cen9. The Wilcoxon test was used to determine the significance of the differences between these two regions.
(TIF)

S24 Fig Correlation between changes in chromatin accessibility intensities and histone modification intensities in the centromere region of Ga chromosome 9 in IL9. Each point represents a 5 kb window. Pearson correlation was calculated between chromatin accessibility changes and histone modification changes.
(TIF)

S25 Fig Distribution density of Non-B-form DNA across Ga chromosome 9. Ga chromosome 9 was divided into non-overlapping 200 kb windows, and the density of Non-B-form DNA in each window was plotted as a line graph. The centromeric region is marked with purple dashed line. A permutation test (n = 1000) was used to determine if the density of Non-B-form DNA in the centromeric region was higher than in non-centromeric regions: **P < 0.01, *P < 0.05, n.s., not significant.
(TIF)

S26 Fig Identification of Non-B-form DNA on Ga chromosome 11. (A) The number of Non-B-form DNA on Ga chromosome 11. A-phased DNA Repeat (APR), Direct Repeat (DR), G-quadruplex (GQ), Inverted Repeats (IR), Mirror Repeat (MR), Short Tandem Repeat (STR), and Z-DNA motifs (Z). (B) Coverage of Non-B-form DNA on Ga chromosome 11. (C) Distribution density of Non-B-form DNA across Ga chromosome 11. Ga chromosome 11 was divided into non-overlapping 200 kb windows, and the density of Non-B-form DNA in each window was plotted as a line graph. The centromeric region is marked with purple dashed line. A permutation test (n = 1000) was used to determine if the density of Non-B-form DNA in the centromeric region was higher than in non-centromeric regions: **P < 0.01. (D) Distribution of CENH3 ChIP-seq read signals on non-B-form DNA. (E) Bar graph showing the density (numbers per 100 kb) of APR in the centromere variable region (R1) and stable region (R2). (F) Box plots displaying the Gibbs free energy in the R1, R2, and non-centromeric regions. The Wilcoxon test was used to determine the significance of differences between these regions.
(TIF)

S27 Fig Distribution density of Non-B-form DNA across Ga chromosome 6. Ga chromosome 6 was divided into non-overlapping 200 kb windows, and the density of Non-B-form DNA in each window was plotted as a line graph. The centromeric region is marked with purple dashed line. A permutation test (n = 1000) was used to determine if the density of Non-B-form DNA in the centromeric region was higher than in non-centromeric regions: **P < 0.01.
(TIF)

S28 Fig Identification of TADs on Ga chromosome 9. TADs, identified as regions of high chromatin interactions, are outlined by black triangles for each biological replicate.
(TIF)

S29 Fig IGV snapshots showing the centromeres and CENH3 binding subdomains on each chromosome of Gh. The CENH3 ChIP-seq track displays the alignment of ChIP-seq reads, with bars in the centromere track indicating centromeric regions. The CENH3 binding subdomains track shows peaks identified by the MACS2 software, representing the CENH3 binding subdomains.
(PDF)

S1 Table Summary of data generated in this study. (PDF)

S2 Table Centromere positions for each chromosome of *G. anomalum.* (PDF)
