# Supplementary figures and images for: Centromere-size reduction and chromatin state dynamics following intergenomic hybridization in cotton
Source: PLoS Genet. 2025 May 2;21(5):e1011689. doi: 10.1371/journal.pgen.1011689 (PMC12068715; doi:10.1371/journal.pgen.1011689)

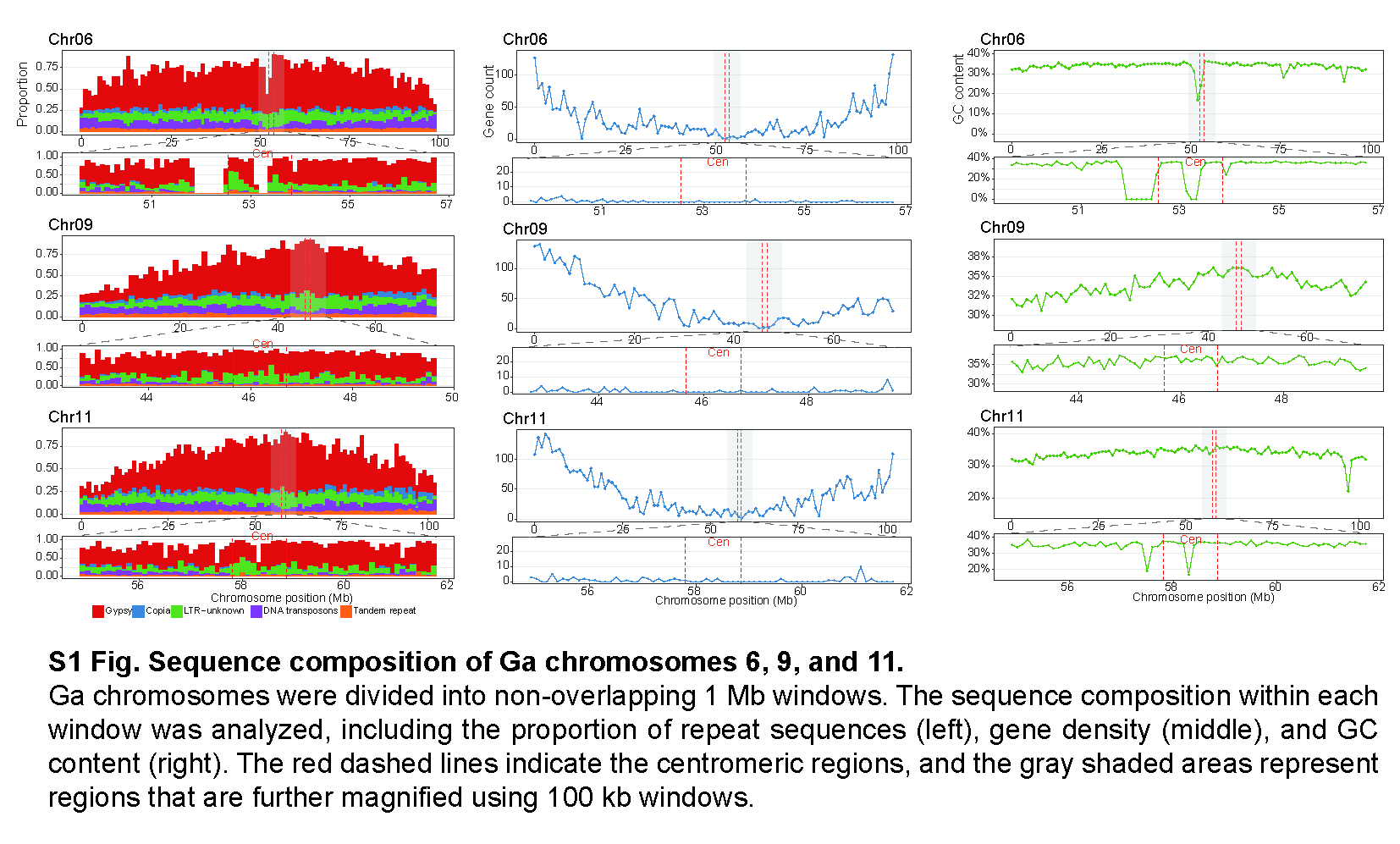

Supplement: S1 Fig — Ga chromosomes were divided into non-overlapping 1 Mb windows. The sequence composition within each window was analyzed, including the proportion of repeat sequences (left), gene density (middle), and GC content (right). The red dashed lines indicate the centromeric regions, and the gray shaded areas represent regions that are further magnified using 100 kb windows. (TIF) [file pgen.1011689.s001.tif]

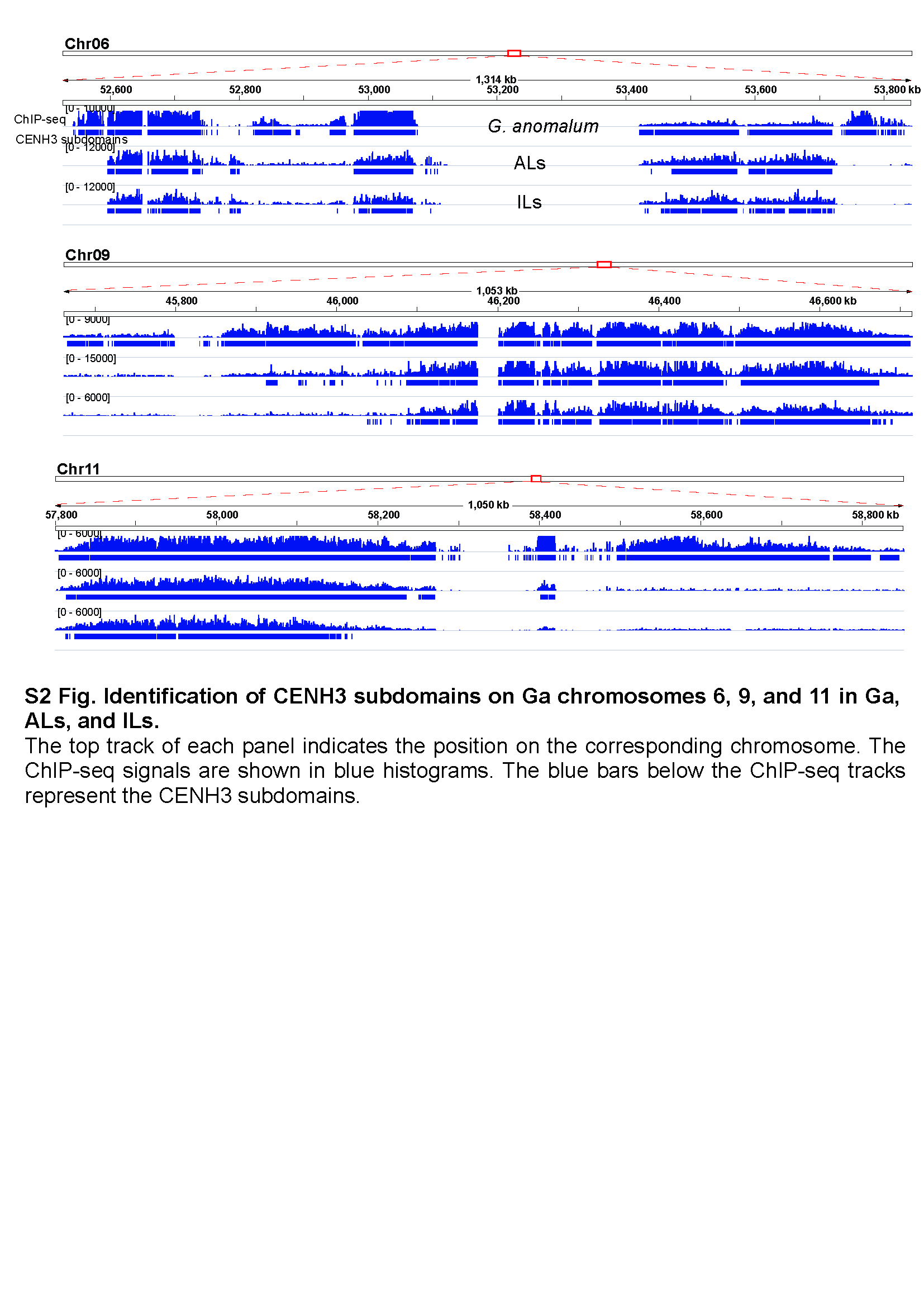

Supplement: S2 Fig — The top track of each panel indicates the position on the corresponding chromosome. The ChIP-seq signals are shown in blue histograms. The blue bars below the ChIP-seq tracks represent the CENH3 subdomains. (TIF) [file pgen.1011689.s002.tif]

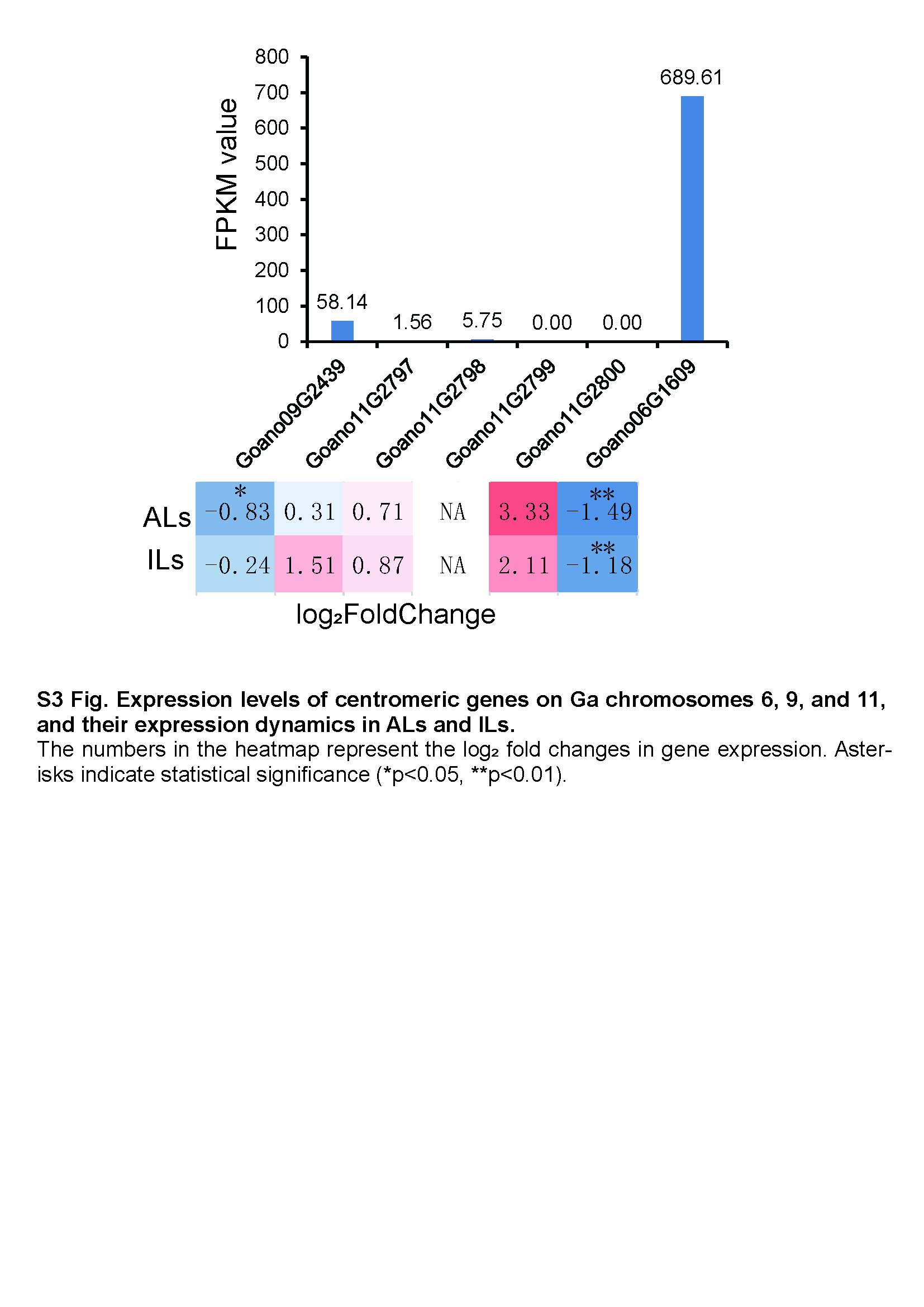

Supplement: S3 Fig — The numbers in the heatmap represent the log2 fold changes in gene expression. Asterisks indicate statistical significance (*p < 0.05, **p < 0.01). (TIF) [file pgen.1011689.s003.tif]

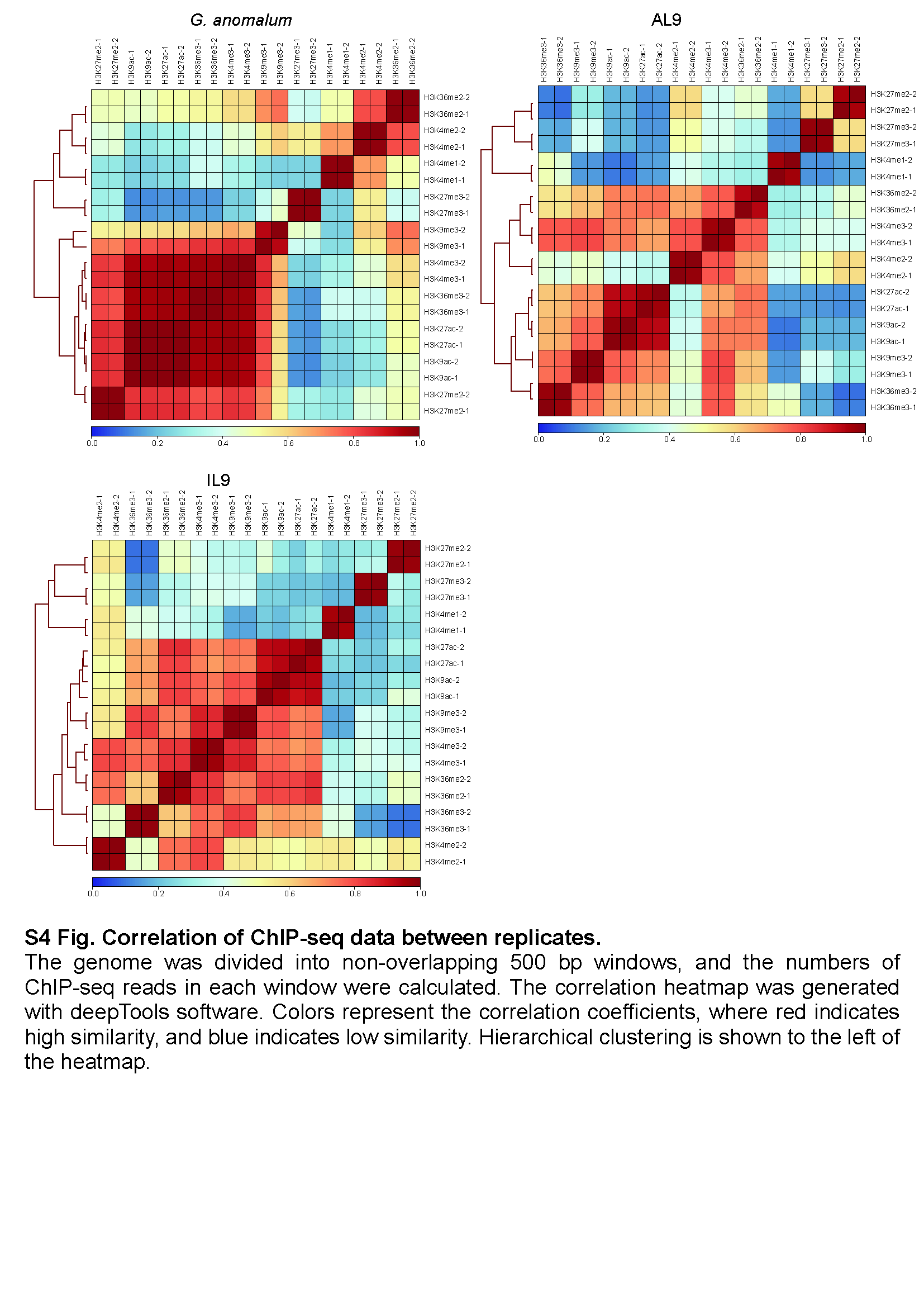

Supplement: S4 Fig — The genome was divided into non-overlapping 500 bp windows, and the numbers of ChIP-seq reads in each window were calculated. The correlation heatmap was generated with deepTools software. Colors represent the correlation coefficients, where red indicates high similarity, and blue indicates low similarity. Hierarchical clustering is shown to the left of the heatmap. (TIF) [file pgen.1011689.s004.tif]

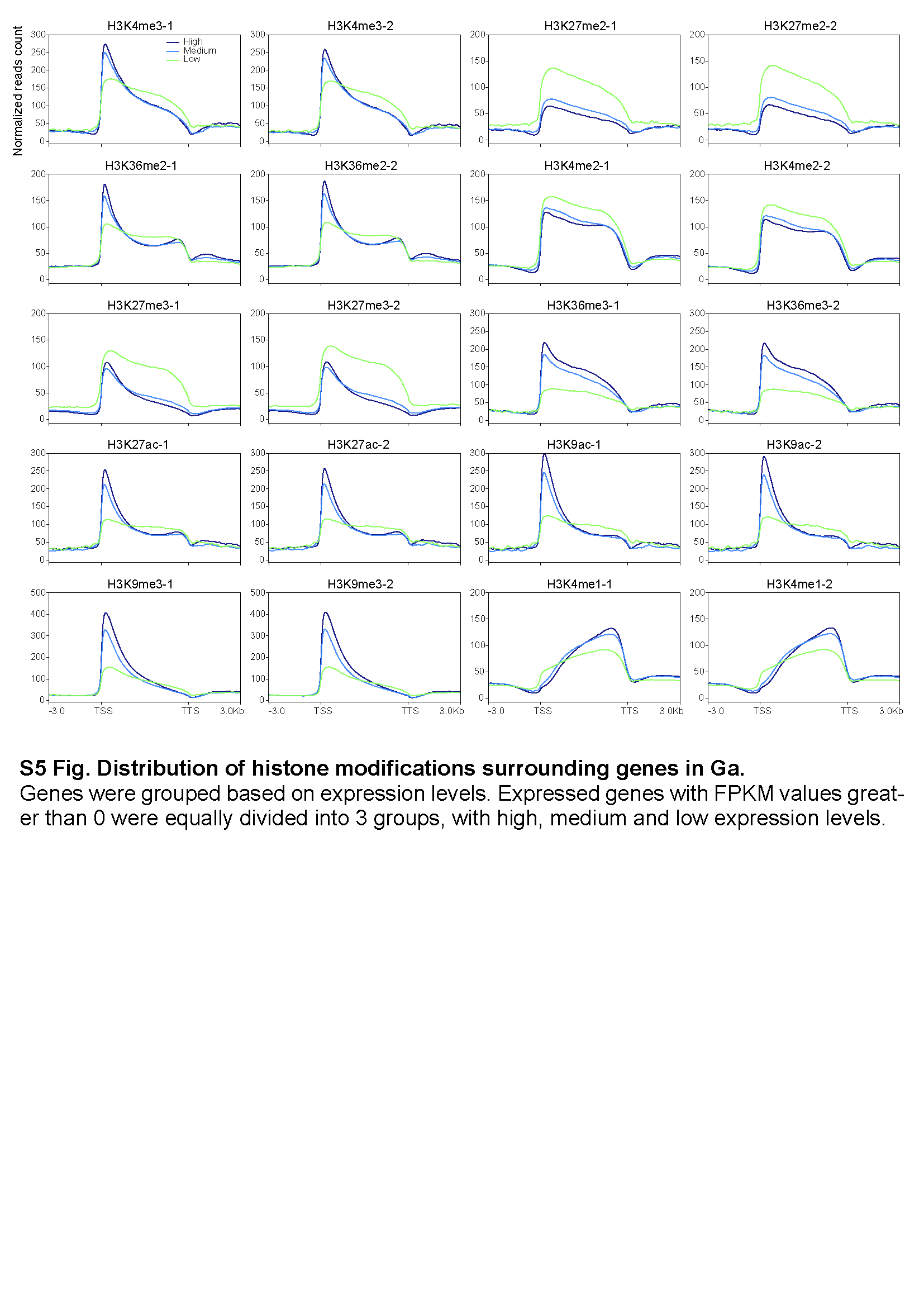

Supplement: S5 Fig — Genes were grouped based on expression levels. Expressed genes with FPKM values greater than 0 were equally divided into 3 groups, with high, medium and low expression levels. (TIF) [file pgen.1011689.s005.tif]

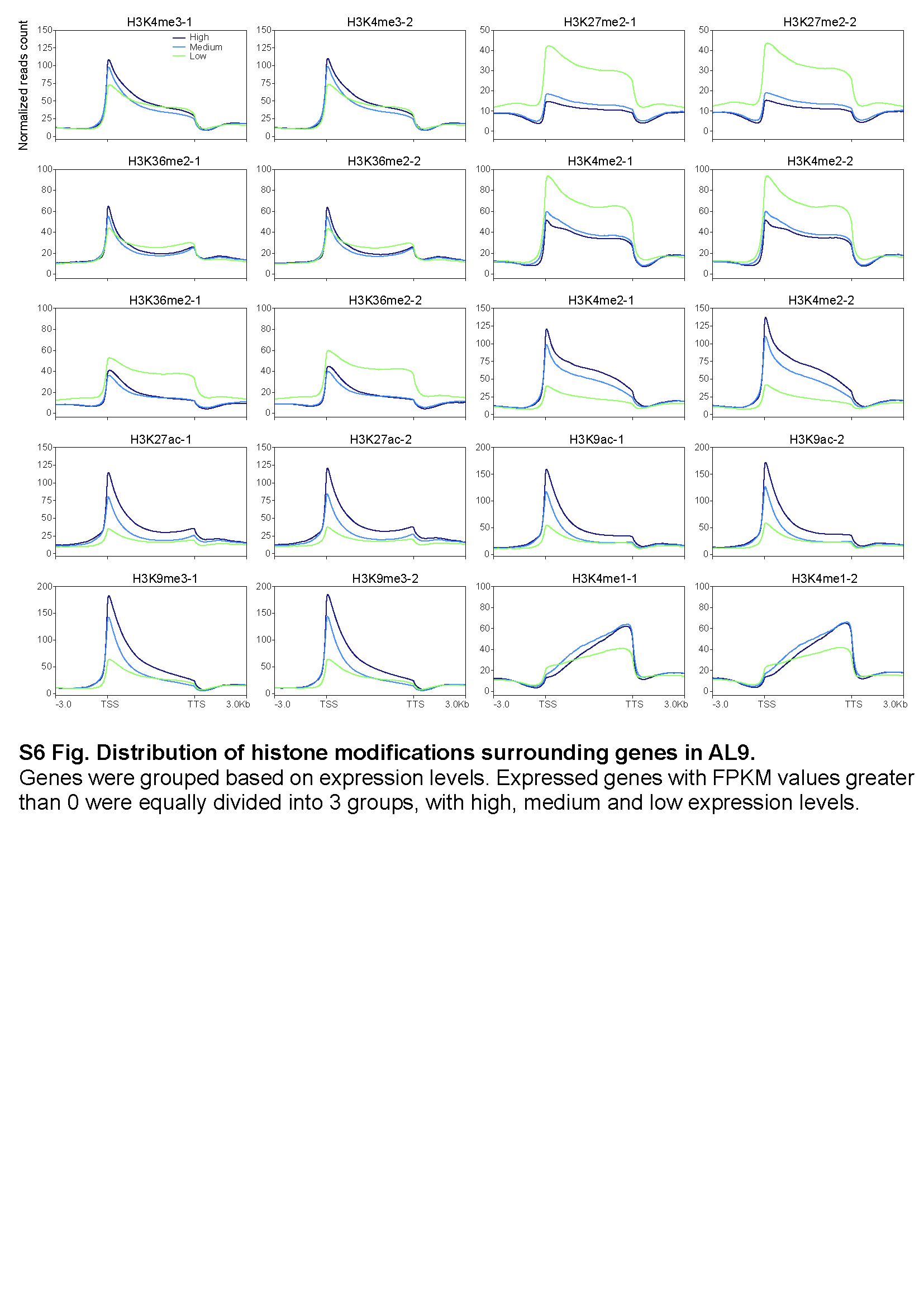

Supplement: S6 Fig — Genes were grouped based on expression levels. Expressed genes with FPKM values greater than 0 were equally divided into 3 groups, with high, medium and low expression levels. (TIF) [file pgen.1011689.s006.tif]

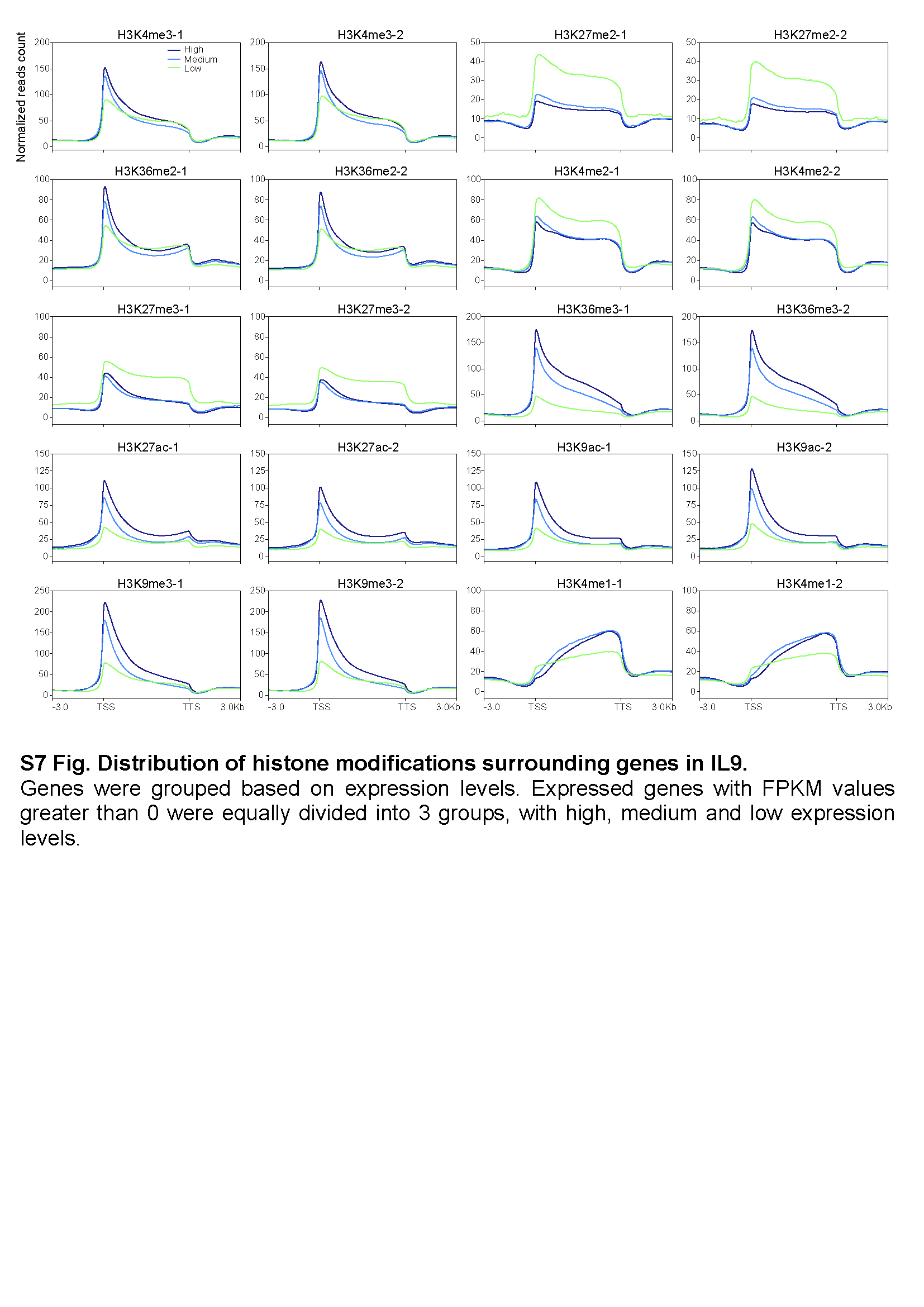

Supplement: S7 Fig — Genes were grouped based on expression levels. Expressed genes with FPKM values greater than 0 were equally divided into 3 groups, with high, medium and low expression levels. (TIF) [file pgen.1011689.s007.tif]

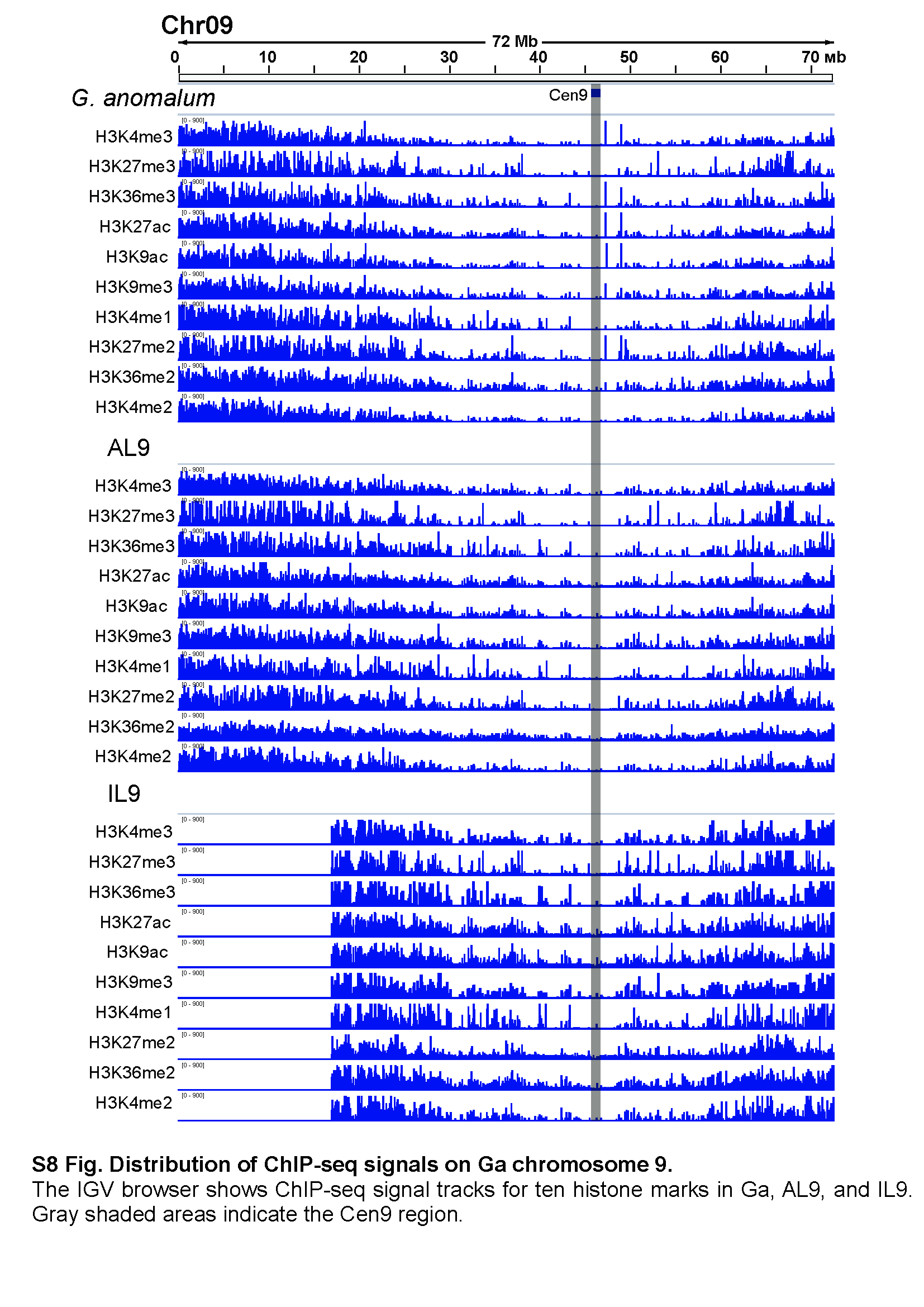

Supplement: S8 Fig — The IGV browser shows ChIP-seq signal tracks for ten histone marks in Ga, AL9, and IL9. Gray shaded areas indicate the Cen9 region. (TIF) [file pgen.1011689.s008.tif]

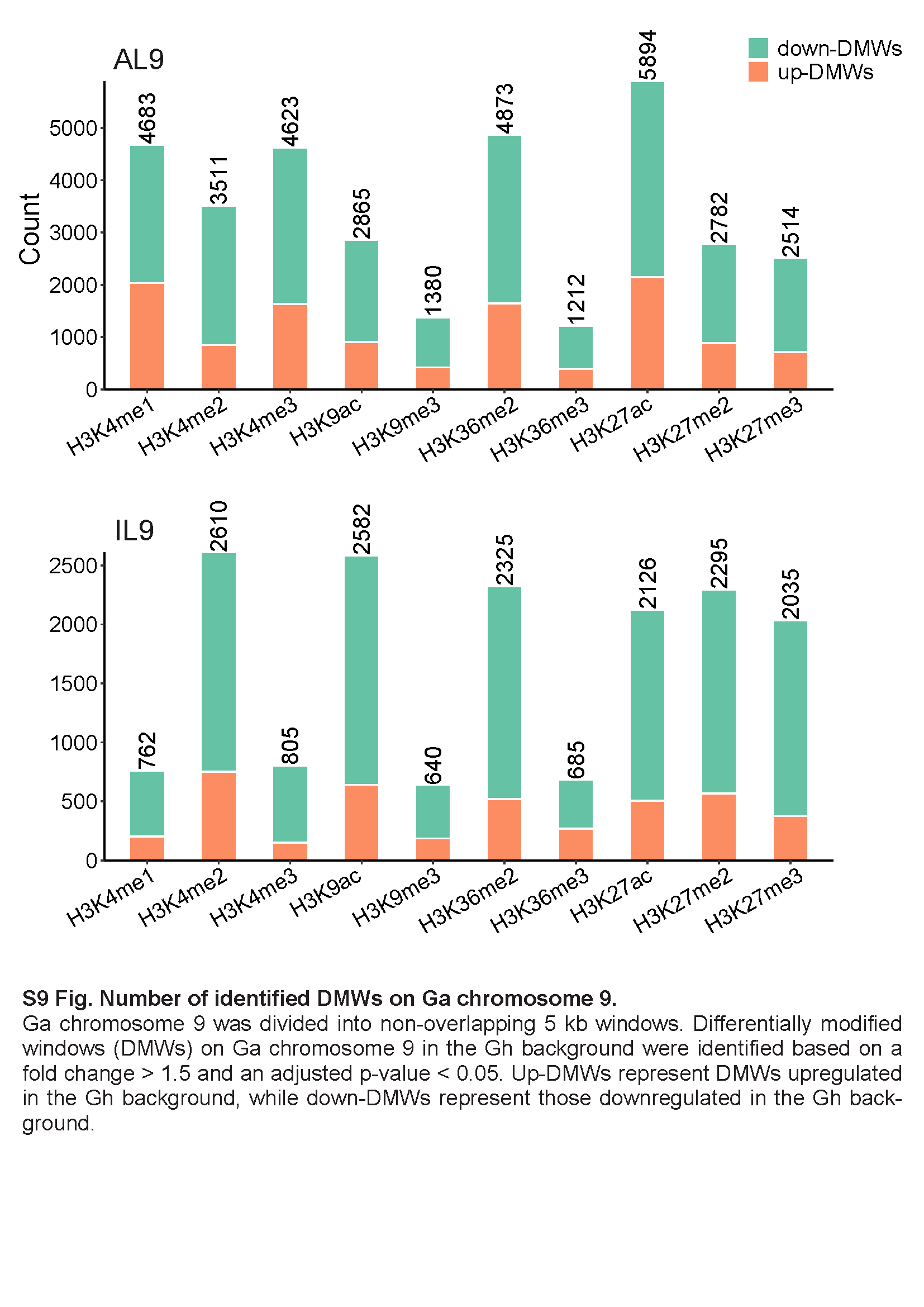

Supplement: S9 Fig — Ga chromosome 9 was divided into non-overlapping 5 kb windows. Differentially modified windows (DMWs) on Ga chromosome 9 in the Gh background were identified based on a fold change > 1.5 and an adjusted p-value < 0.05. Up-DMWs represent DMWs upregulated in the Gh background, while down-DMWs represent those downregulated in the Gh background. (TIF) [file pgen.1011689.s009.tif]

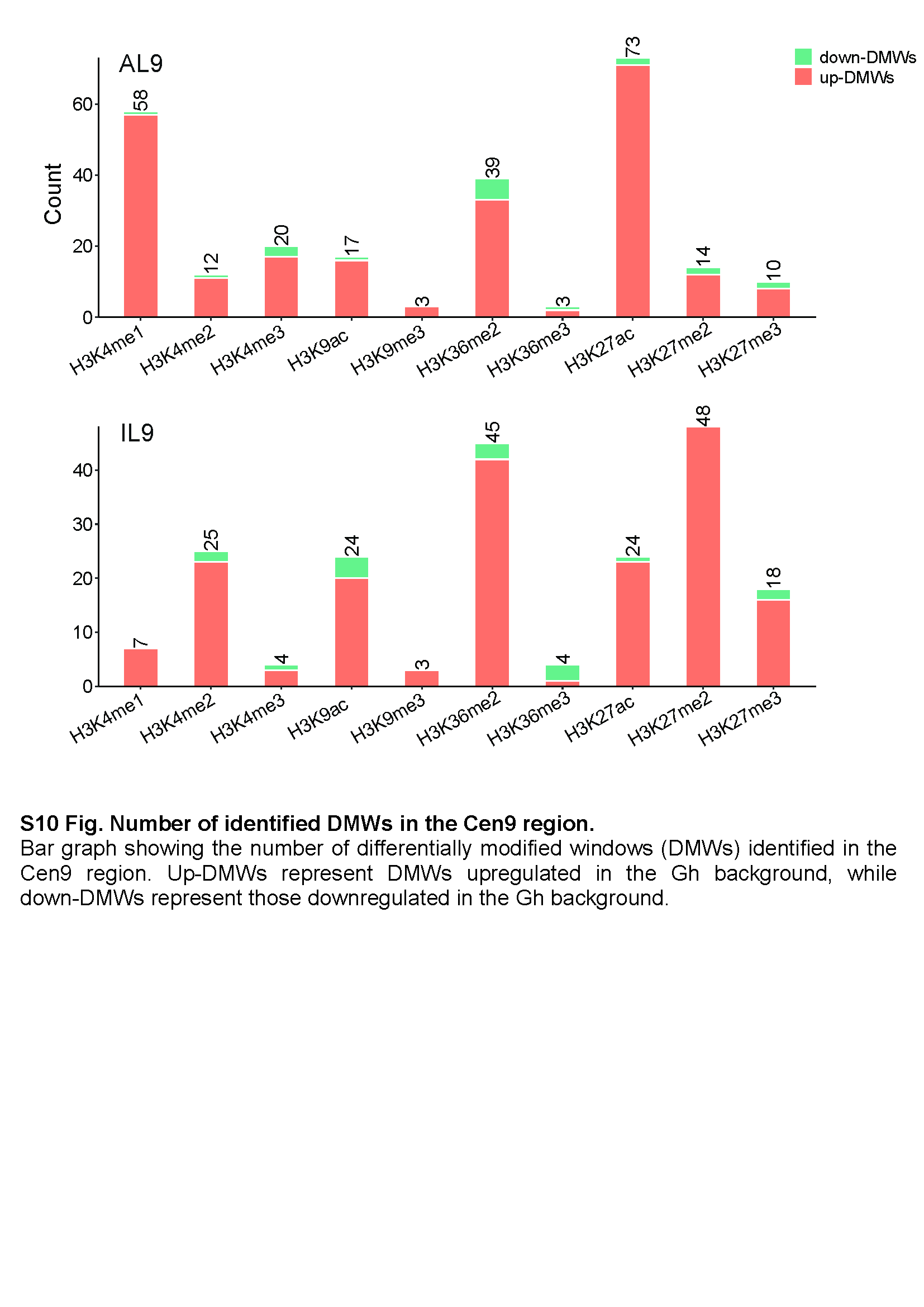

Supplement: S10 Fig — Bar graph showing the number of differentially modified windows (DMWs) identified in the Cen9 region. Up-DMWs represent DMWs upregulated in the Gh background, while down-DMWs represent those downregulated in the Gh background. (TIF) [file pgen.1011689.s010.tif]

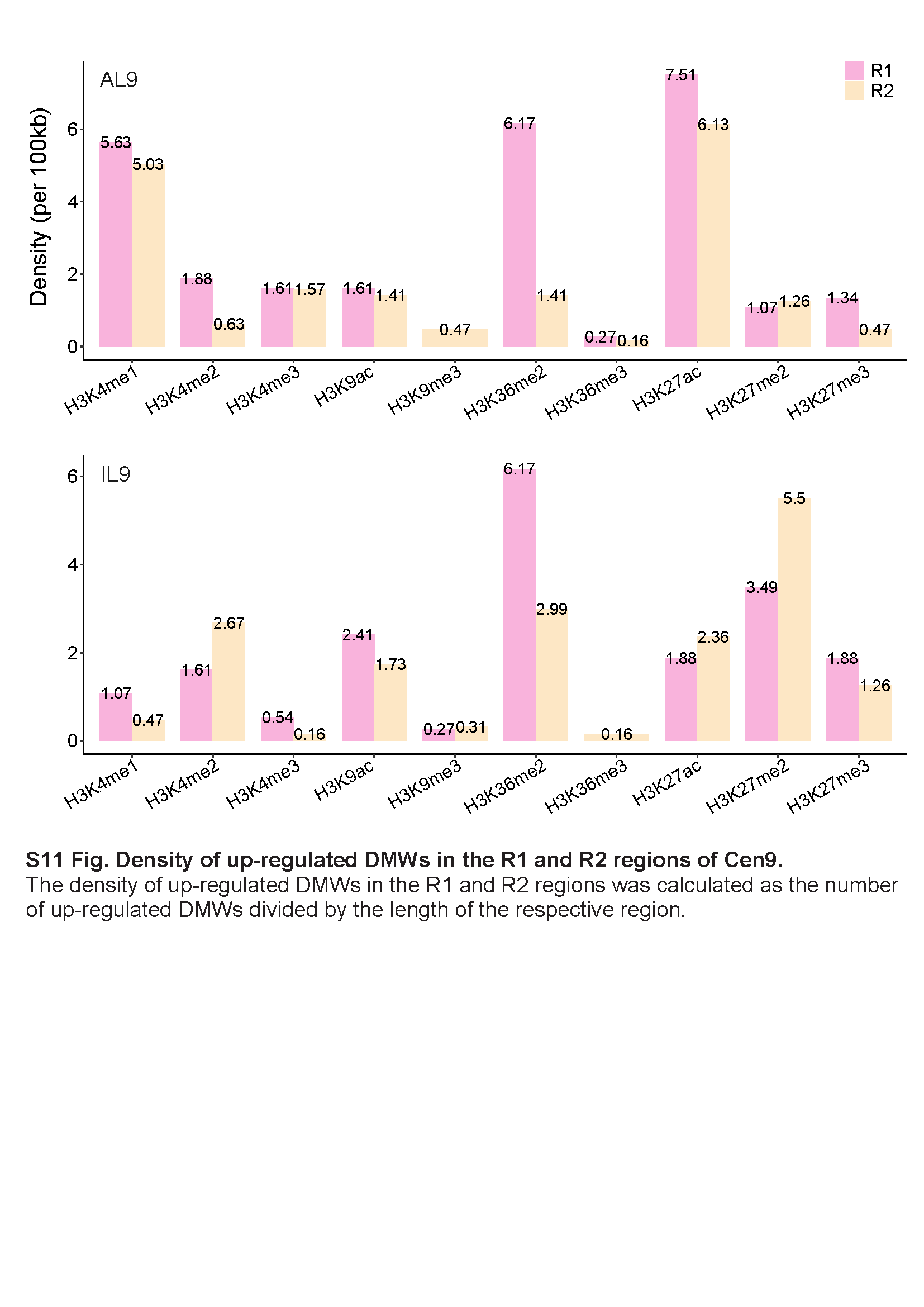

Supplement: S11 Fig — The density of up-regulated DMWs in the R1 and R2 regions was calculated as the number of up-regulated DMWs divided by the length of the respective region. (TIF) [file pgen.1011689.s011.tif]

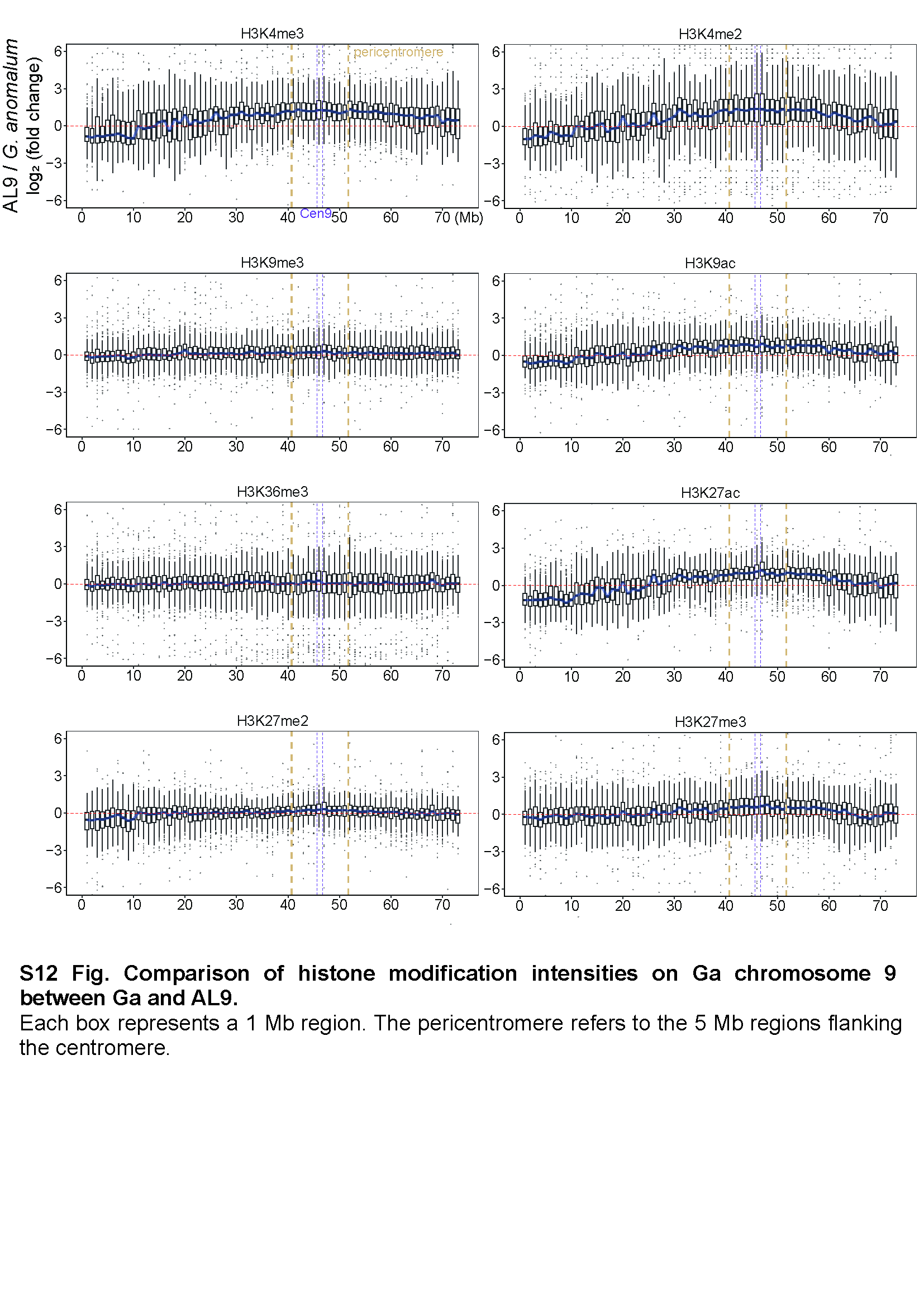

Supplement: S12 Fig — Each box represents a 1 Mb region. The pericentromere refers to the 5 Mb regions flanking the centromere. (TIF) [file pgen.1011689.s012.tif]

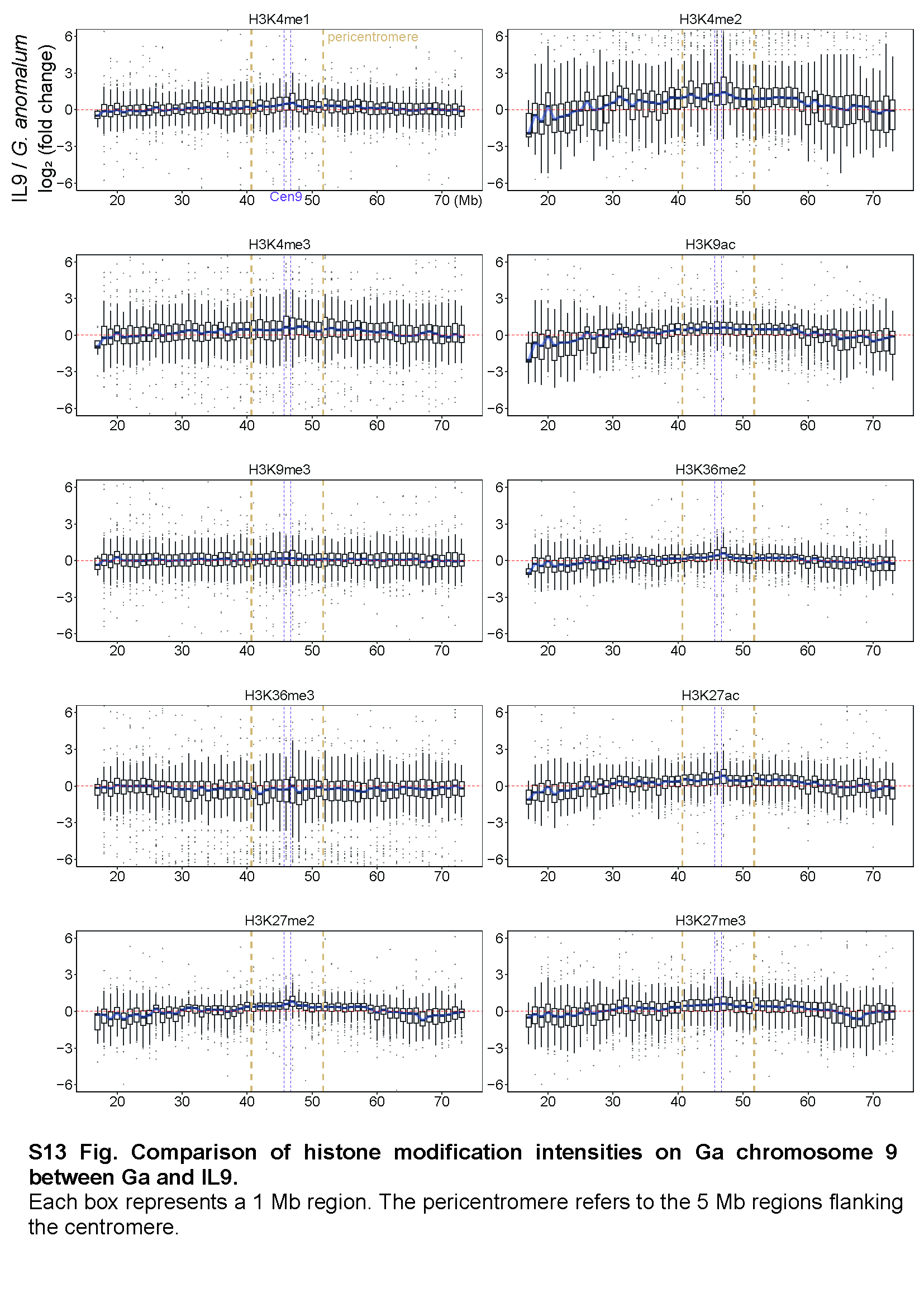

Supplement: S13 Fig — Each box represents a 1 Mb region. The pericentromere refers to the 5 Mb regions flanking the centromere. (TIF) [file pgen.1011689.s013.tif]

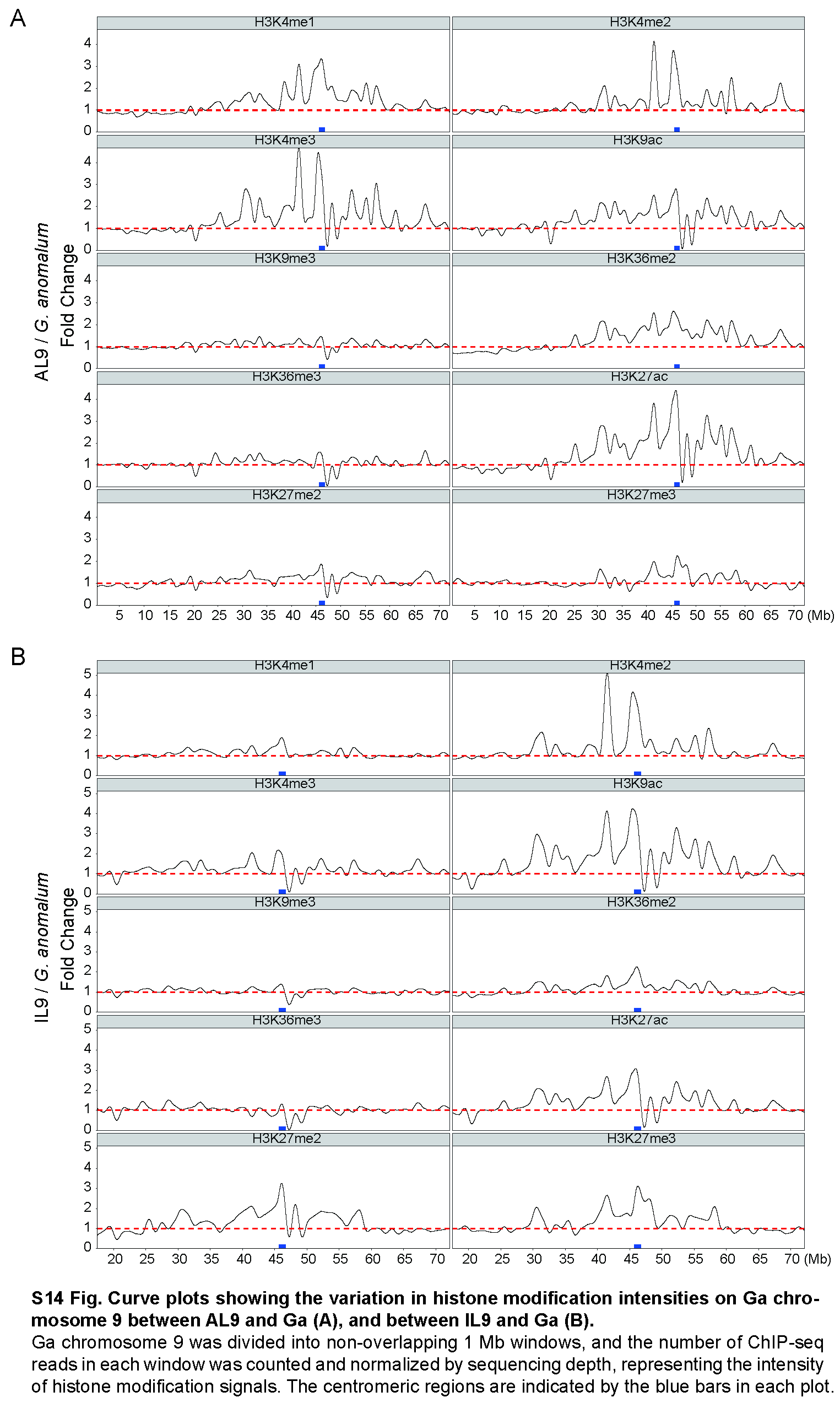

Supplement: S14 Fig — Ga chromosome 9 was divided into non-overlapping 1 Mb windows, and the number of ChIP-seq reads in each window was counted and normalized by sequencing depth, representing the intensity of histone modification signals. The centromeric regions are indicated by the blue bars in each plot. (TIF) [file pgen.1011689.s014.tif]

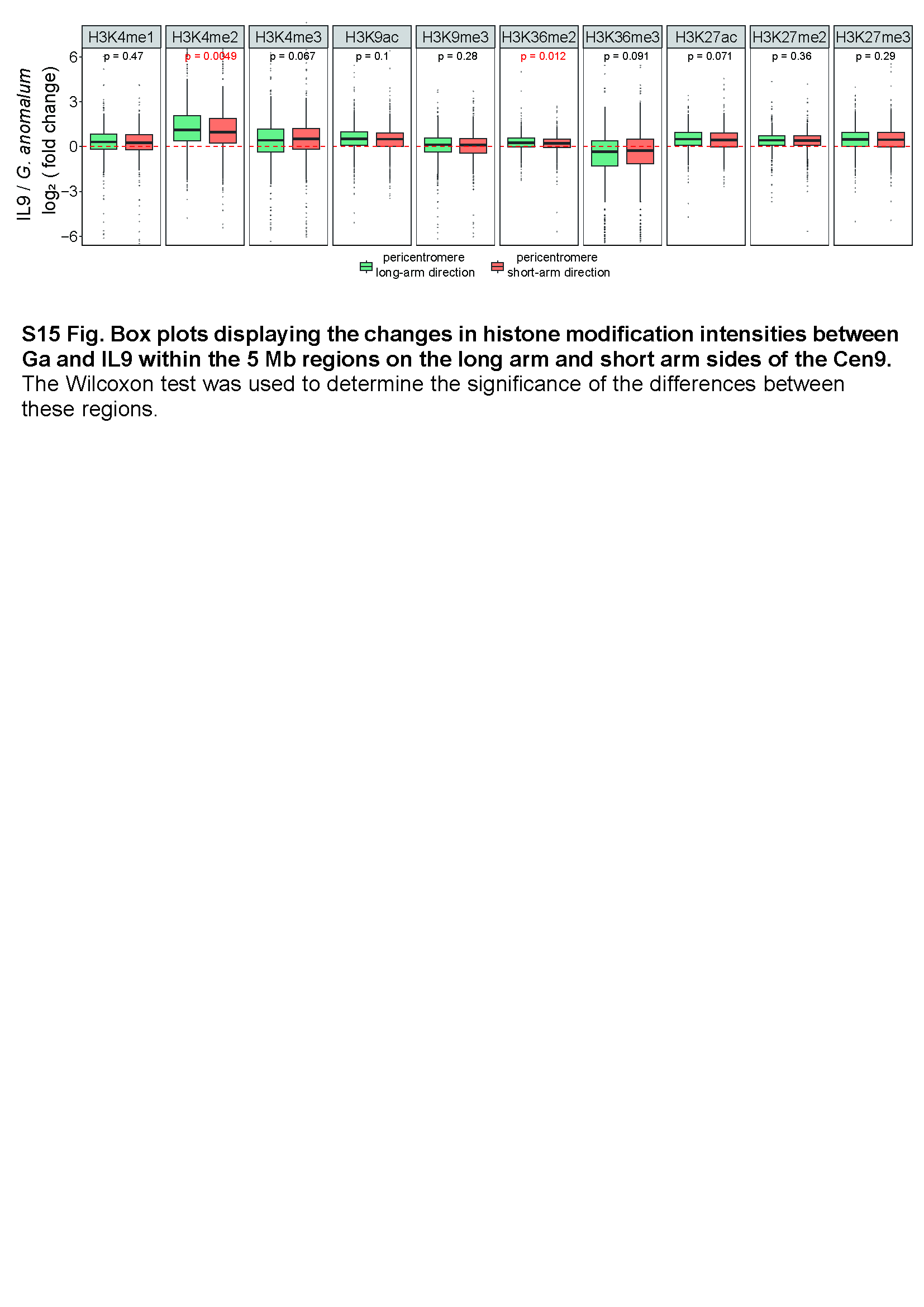

Supplement: S15 Fig — The Wilcoxon test was used to determine the significance of the differences between these regions. (TIF) [file pgen.1011689.s015.tif]

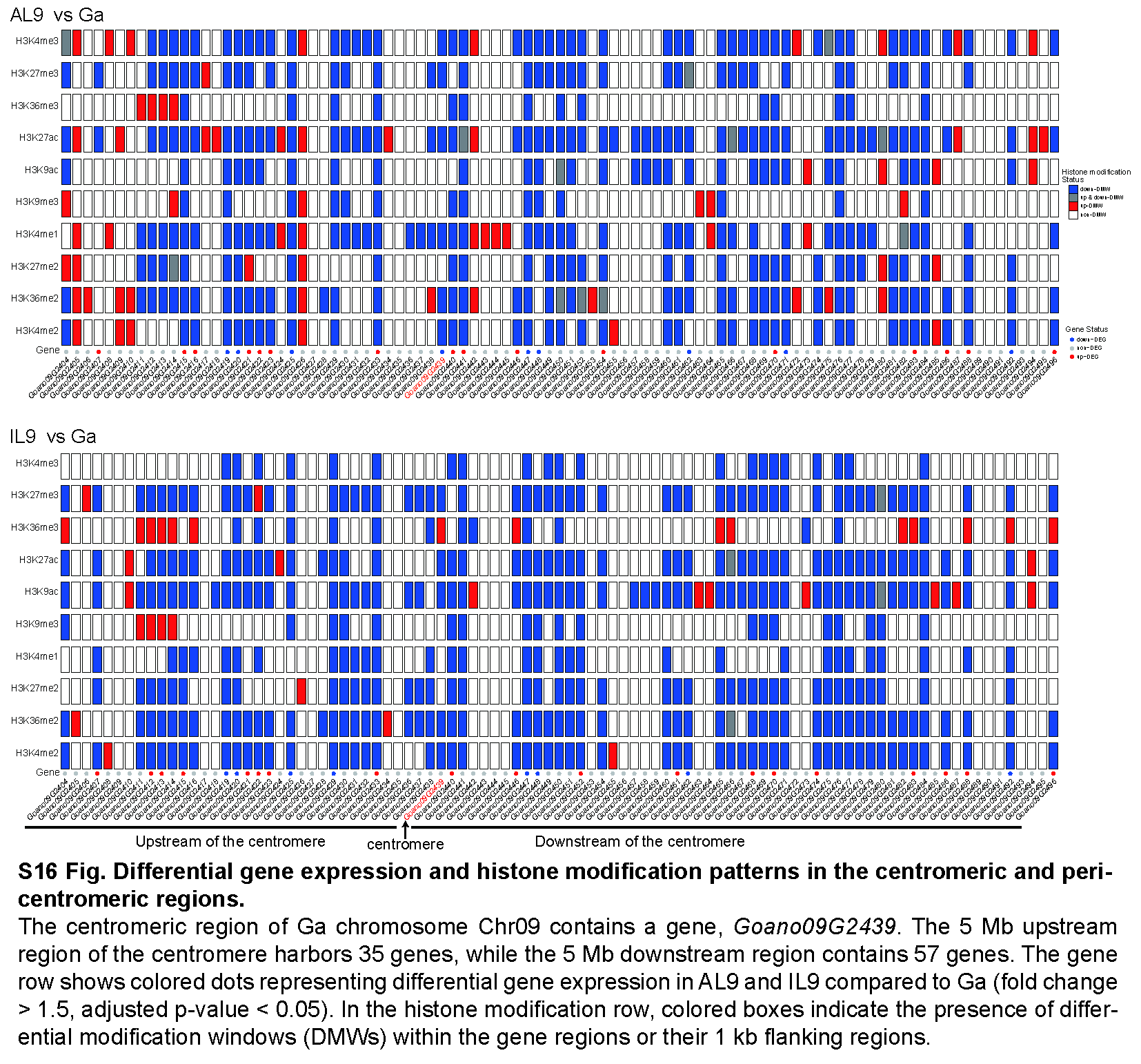

Supplement: S16 Fig — The centromeric region of Ga chromosome Chr09 contains a gene, Goano09G2439. The 5 Mb upstream region of the centromere harbors 35 genes, while the 5 Mb downstream region contains 57 genes. The gene row shows colored dots representing differential gene expression in AL9 and IL9 compared to Ga (fold change > 1.5, adjusted p-value < 0.05). In the histone modification row, colored boxes indicate the presence of differential modification windows (DMWs) within the gene regions or their 1 kb flanking regions. (TIF) [file pgen.1011689.s016.tif]

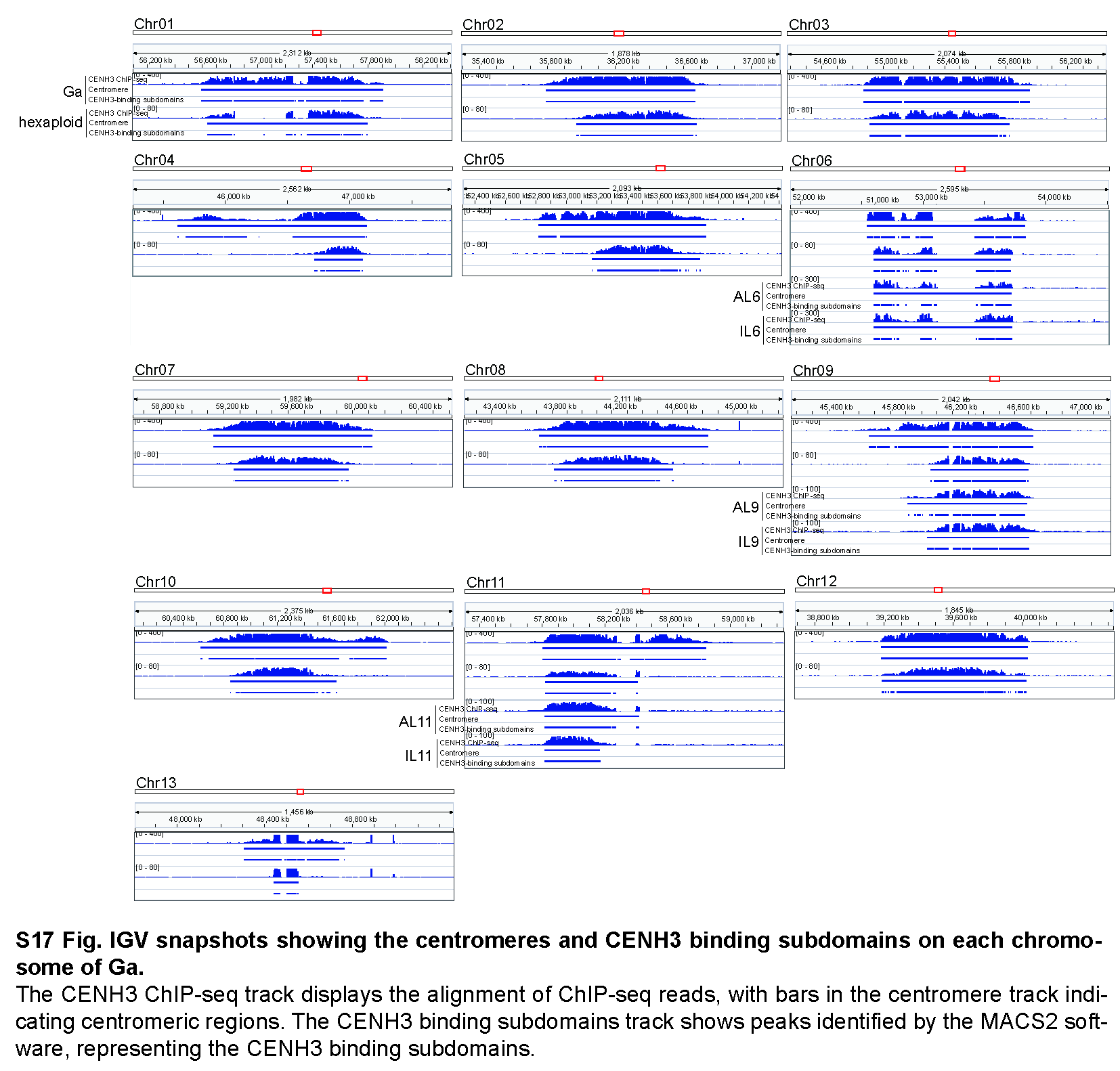

Supplement: S17 Fig — The CENH3 ChIP-seq track displays the alignment of ChIP-seq reads, with bars in the centromere track indicating centromeric regions. The CENH3 binding subdomains track shows peaks identified by the MACS2 software, representing the CENH3 binding subdomains. (TIF) [file pgen.1011689.s017.tif]

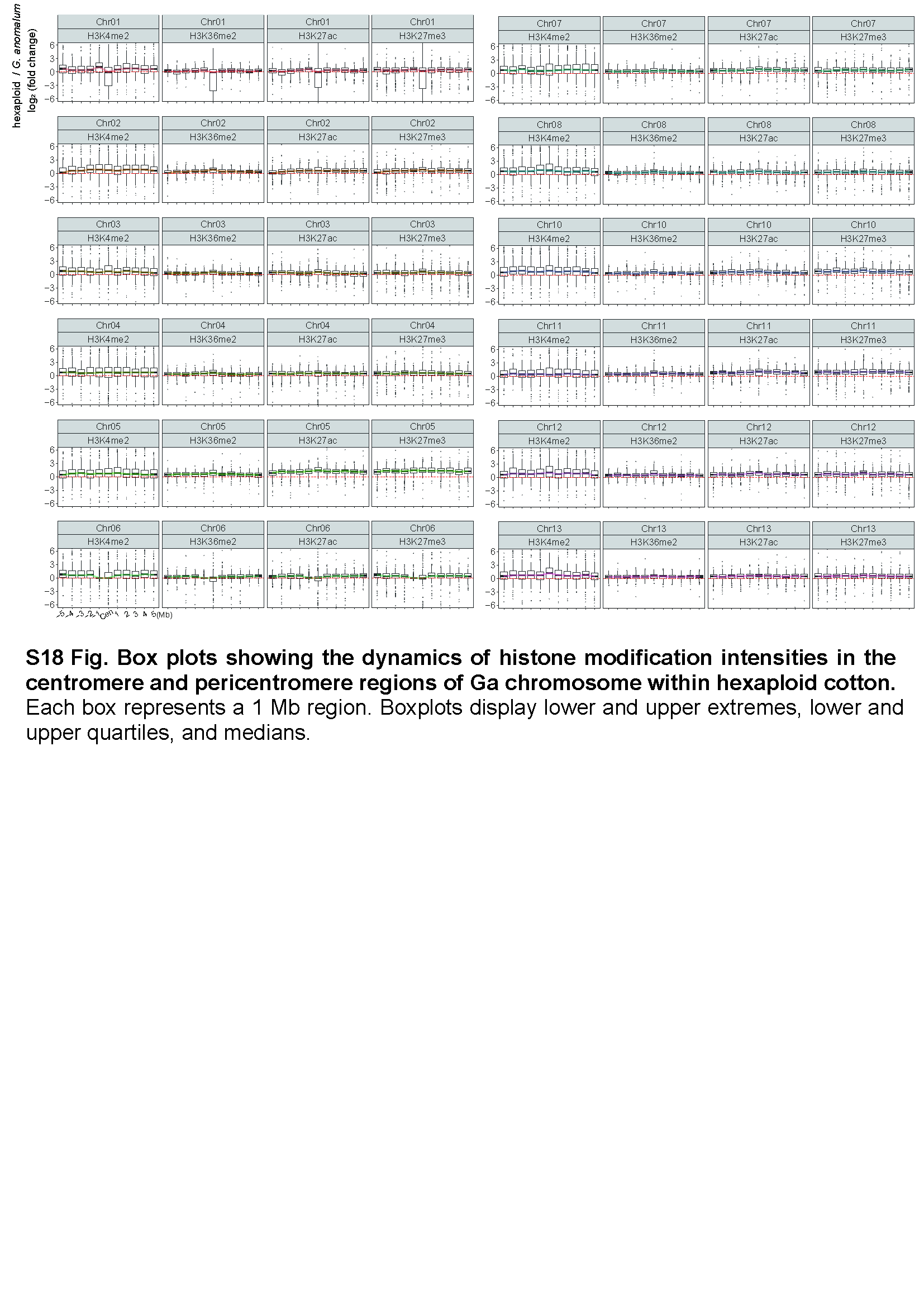

Supplement: S18 Fig — Each box represents a 1 Mb region. Boxplots display lower and upper extremes, lower and upper quartiles, and medians. (TIF) [file pgen.1011689.s018.tif]

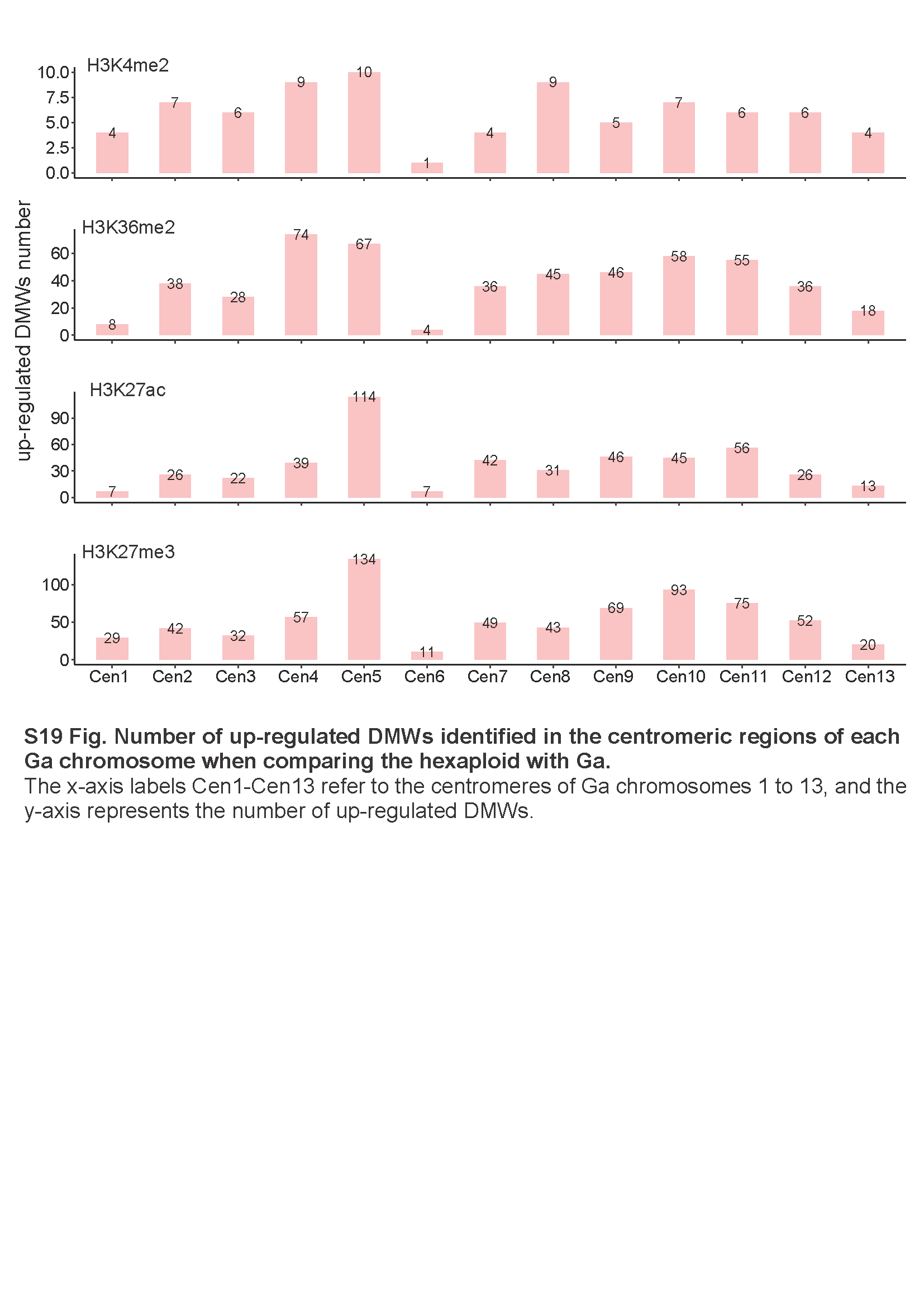

Supplement: S19 Fig — The x-axis labels Cen1-Cen13 refer to the centromeres of Ga chromosomes 1–13, and the y-axis represents the number of up-regulated DMWs. (TIF) [file pgen.1011689.s019.tif]

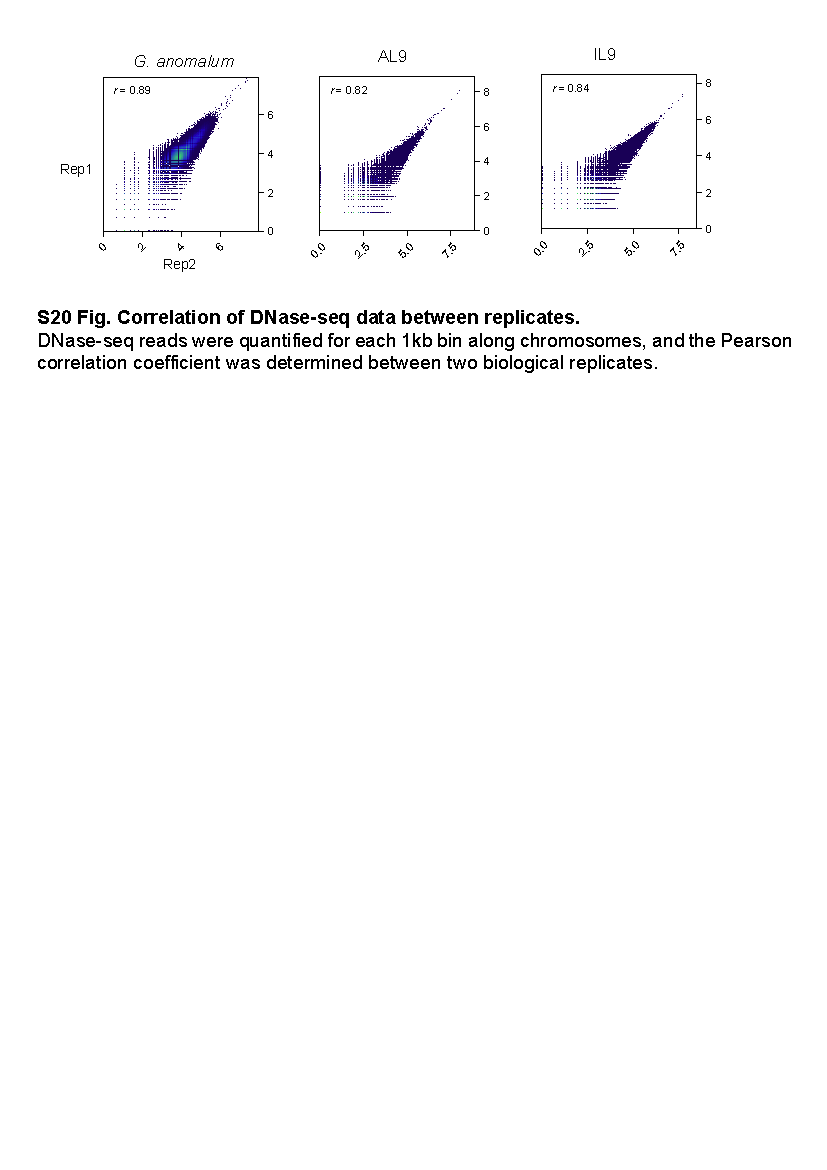

Supplement: S20 Fig — DNase-seq reads were quantified for each 1kb bin along chromosomes, and the Pearson correlation coefficient was determined between two biological replicates. (TIF) [file pgen.1011689.s020.tif]

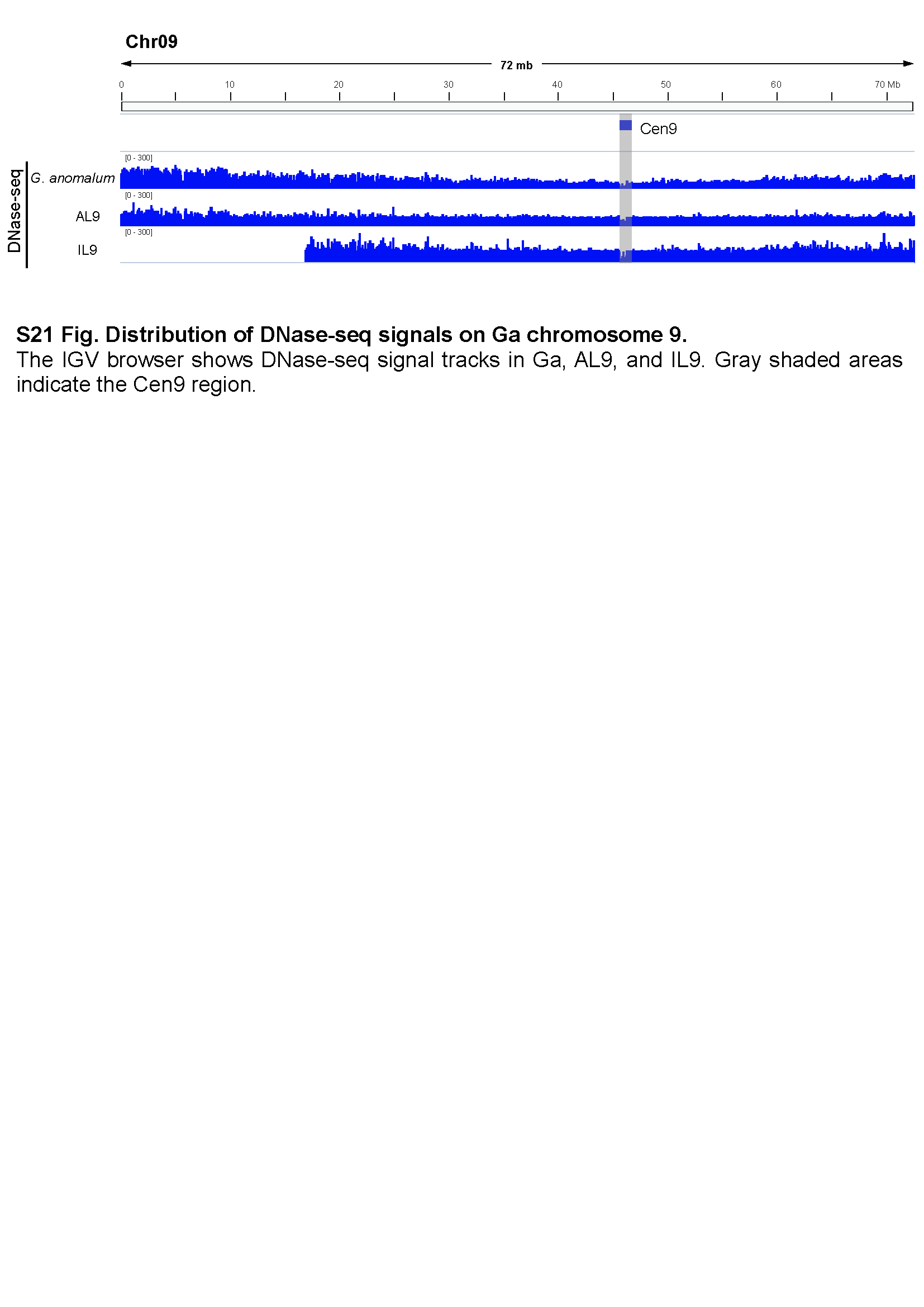

Supplement: S21 Fig — The IGV browser shows DNase-seq signal tracks in Ga, AL9, and IL9. Gray shaded areas indicate the Cen9 region. (TIF) [file pgen.1011689.s021.tif]

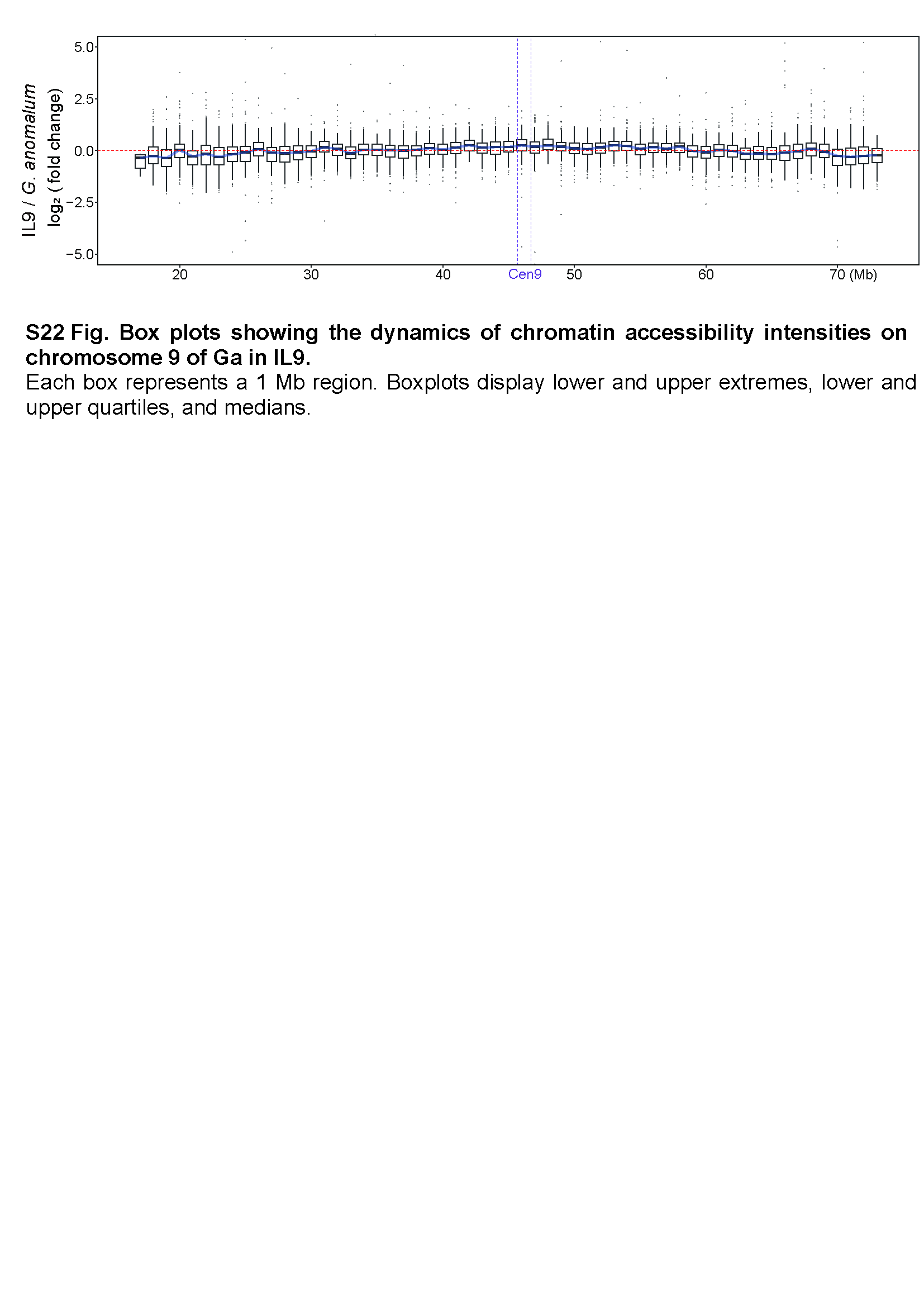

Supplement: S22 Fig — Each box represents a 1 Mb region. Boxplots display lower and upper extremes, lower and upper quartiles, and medians. (TIF) [file pgen.1011689.s022.tif]

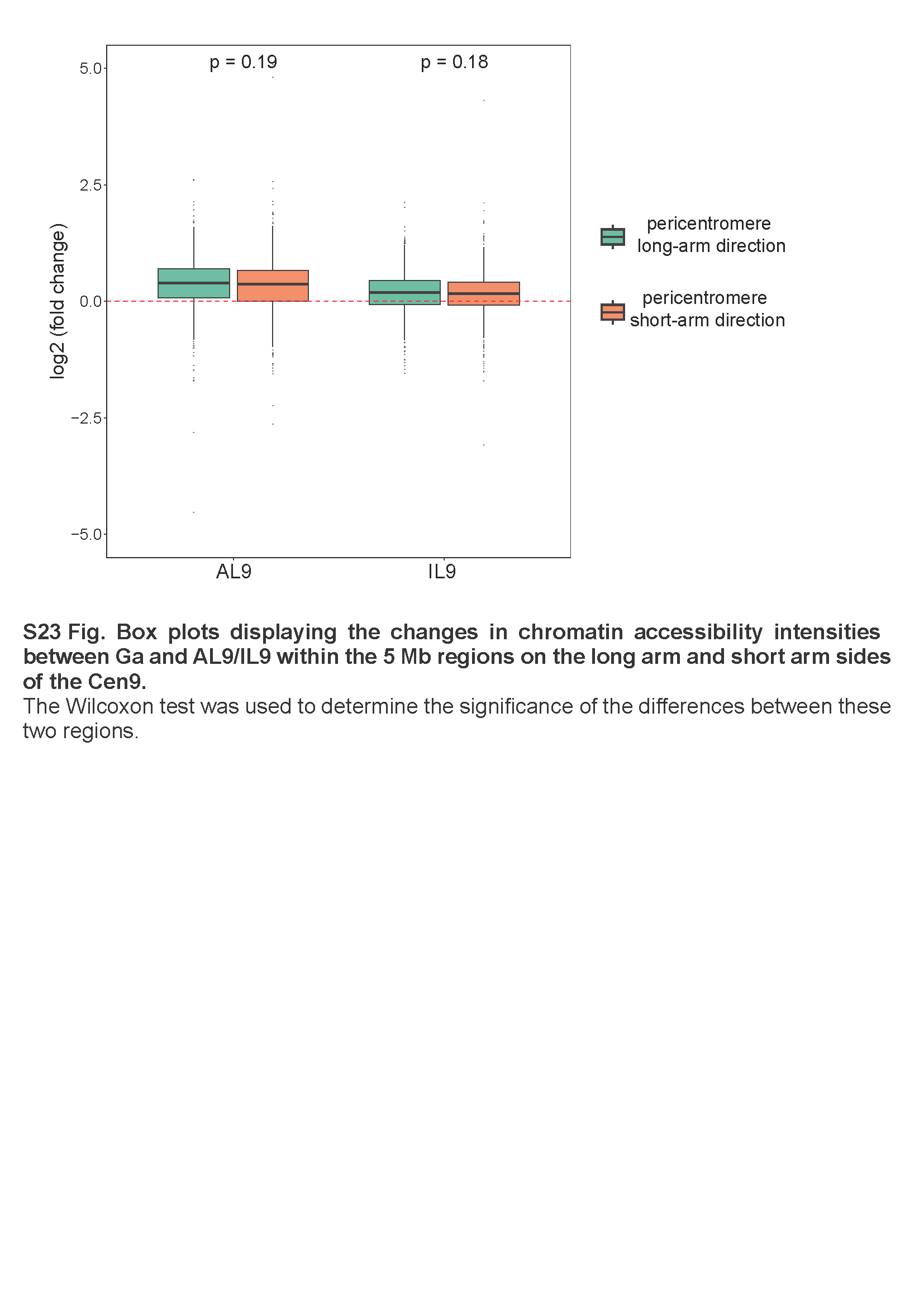

Supplement: S23 Fig — The Wilcoxon test was used to determine the significance of the differences between these two regions. (TIF) [file pgen.1011689.s023.tif]

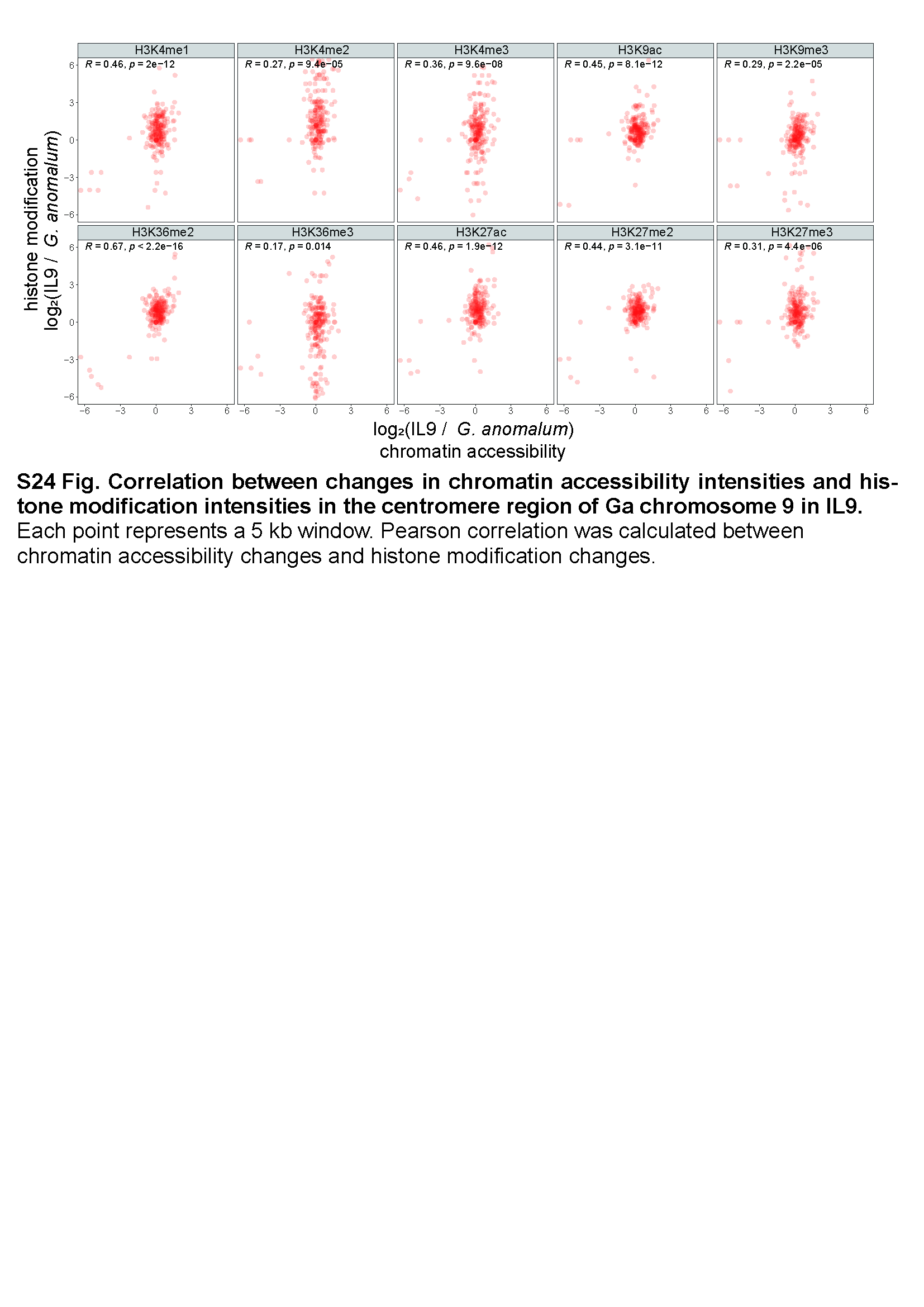

Supplement: S24 Fig — Each point represents a 5 kb window. Pearson correlation was calculated between chromatin accessibility changes and histone modification changes. (TIF) [file pgen.1011689.s024.tif]

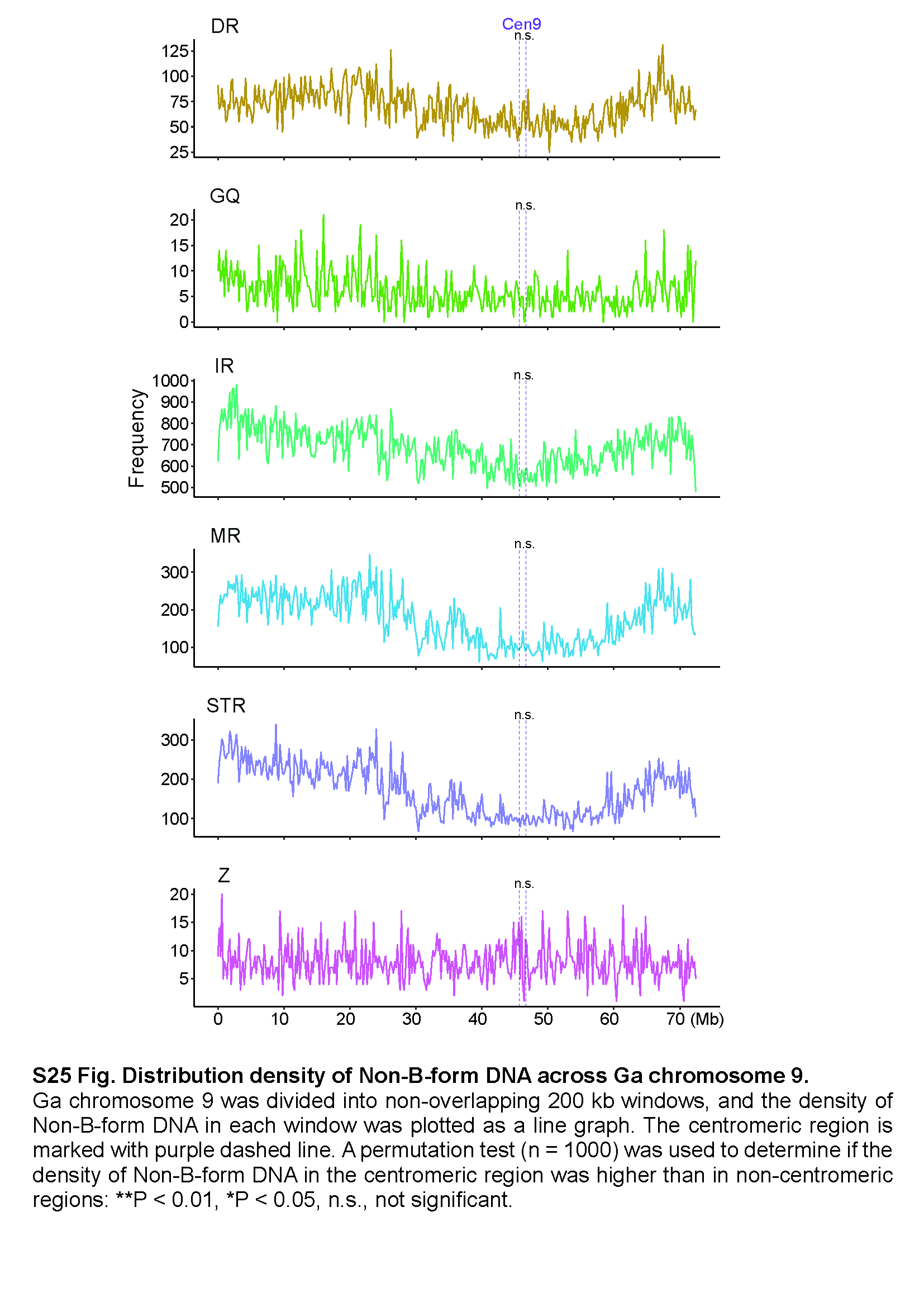

Supplement: S25 Fig — Ga chromosome 9 was divided into non-overlapping 200 kb windows, and the density of Non-B-form DNA in each window was plotted as a line graph. The centromeric region is marked with purple dashed line. A permutation test (n = 1000) was used to determine if the density of Non-B-form DNA in the centromeric region was higher than in non-centromeric regions: **P < 0.01, *P < 0.05, n.s., not significant. (TIF) [file pgen.1011689.s025.tif]

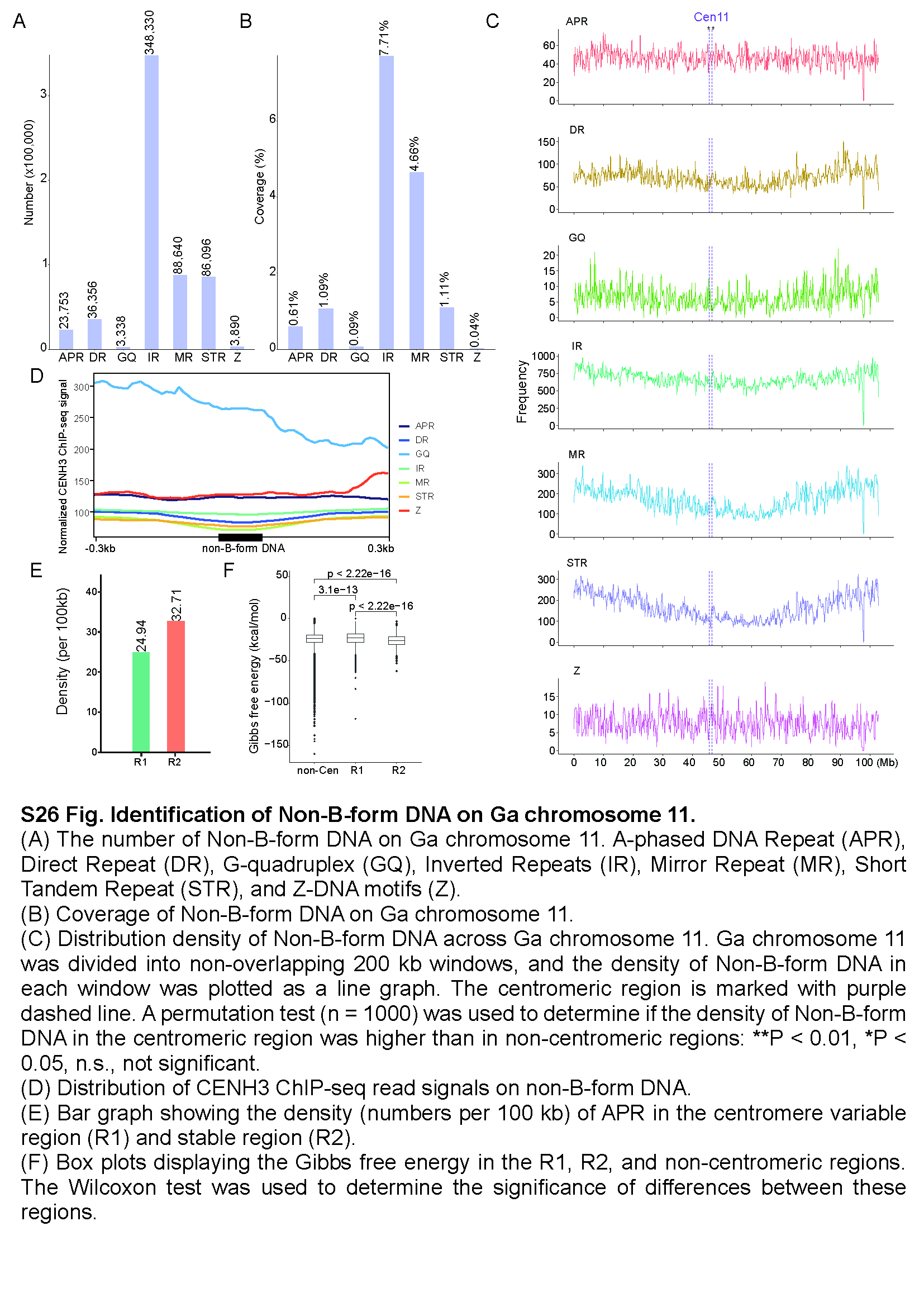

Supplement: S26 Fig — (A) The number of Non-B-form DNA on Ga chromosome 11. A-phased DNA Repeat (APR), Direct Repeat (DR), G-quadruplex (GQ), Inverted Repeats (IR), Mirror Repeat (MR), Short Tandem Repeat (STR), and Z-DNA motifs (Z). (B) Coverage of Non-B-form DNA on Ga chromosome 11. (C) Distribution density of Non-B-form DNA across Ga chromosome 11. Ga chromosome 11 was divided into non-overlapping 200 kb windows, and the density of Non-B-form DNA in each window was plotted as a line graph. The centromeric region is marked with purple dashed line. A permutation test (n = 1000) was used to determine if the density of Non-B-form DNA in the centromeric region was higher than in non-centromeric regions: **P < 0.01. (D) Distribution of CENH3 ChIP-seq read signals on non-B-form DNA. (E) Bar graph showing the density (numbers per 100 kb) of APR in the centromere variable region (R1) and stable region (R2). (F) Box plots displaying the Gibbs free energy in the R1, R2, and non-centromeric regions. The Wilcoxon test was used to determine the significance of differences between these regions. (TIF) [file pgen.1011689.s026.tif]

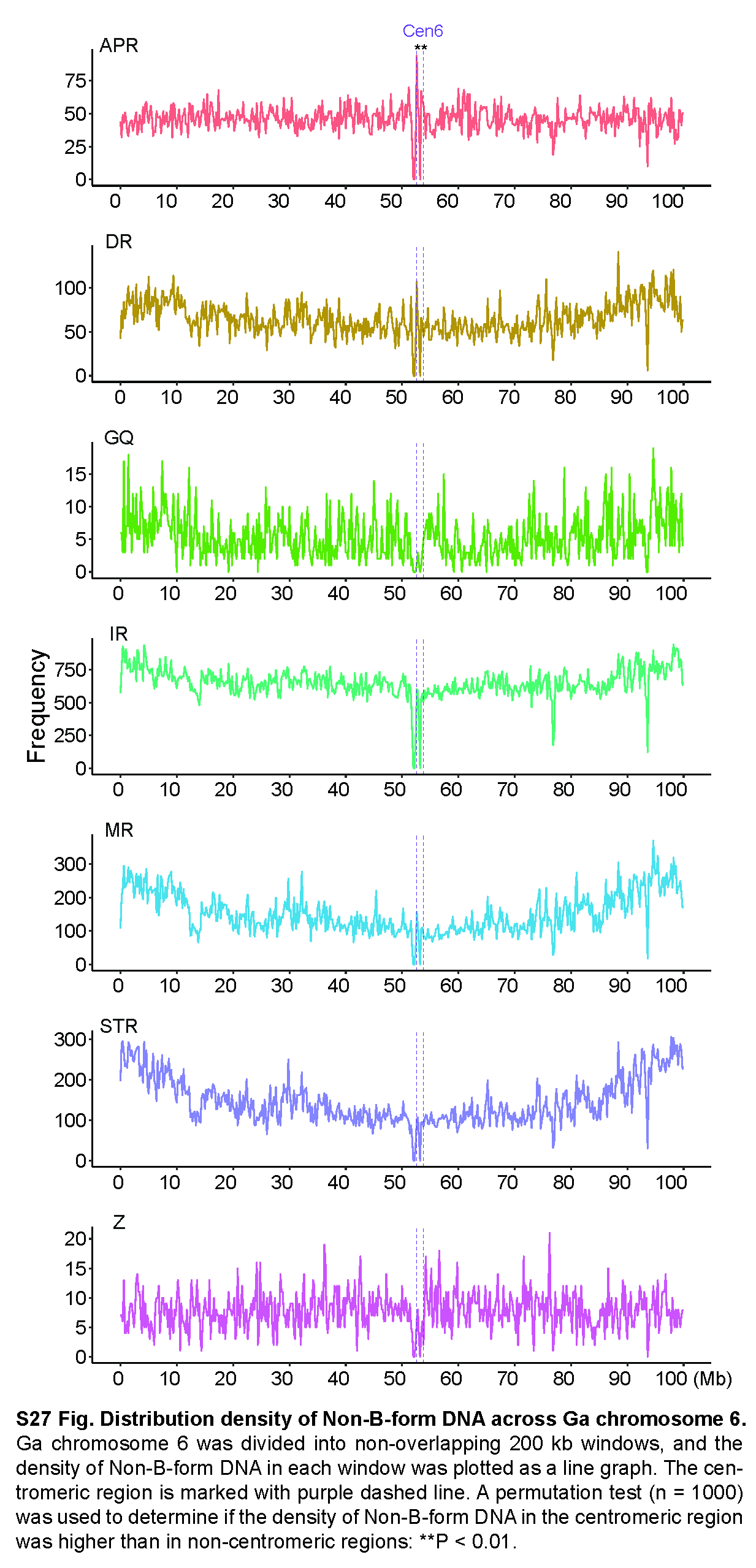

Supplement: S27 Fig — Ga chromosome 6 was divided into non-overlapping 200 kb windows, and the density of Non-B-form DNA in each window was plotted as a line graph. The centromeric region is marked with purple dashed line. A permutation test (n = 1000) was used to determine if the density of Non-B-form DNA in the centromeric region was higher than in non-centromeric regions: **P < 0.01. (TIF) [file pgen.1011689.s027.tif]

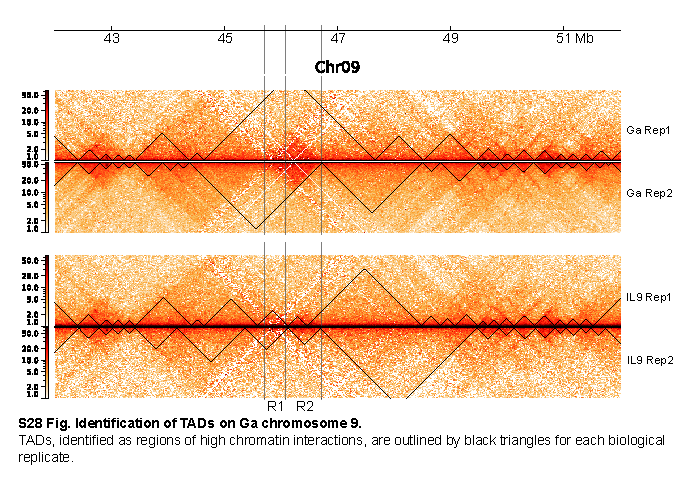

Supplement: S28 Fig — TADs, identified as regions of high chromatin interactions, are outlined by black triangles for each biological replicate. (TIF) [file pgen.1011689.s028.tif]
