# Supplementary material for: Centromere-size reduction and chromatin state dynamics following intergenomic hybridization in cotton
Source: PLoS Genet. 2025 May 2;21(5):e1011689. doi: 10.1371/journal.pgen.1011689 (PMC12068715; doi:10.1371/journal.pgen.1011689)
Supplement: S29 Fig — The CENH3 ChIP-seq track displays the alignment of ChIP-seq reads, with bars in the centromere track indicating centromeric regions. The CENH3 binding subdomains track shows peaks identified by the MACS2 software, representing the CENH3 binding subdomains. (PDF) [file pgen.1011689.s029.pdf]

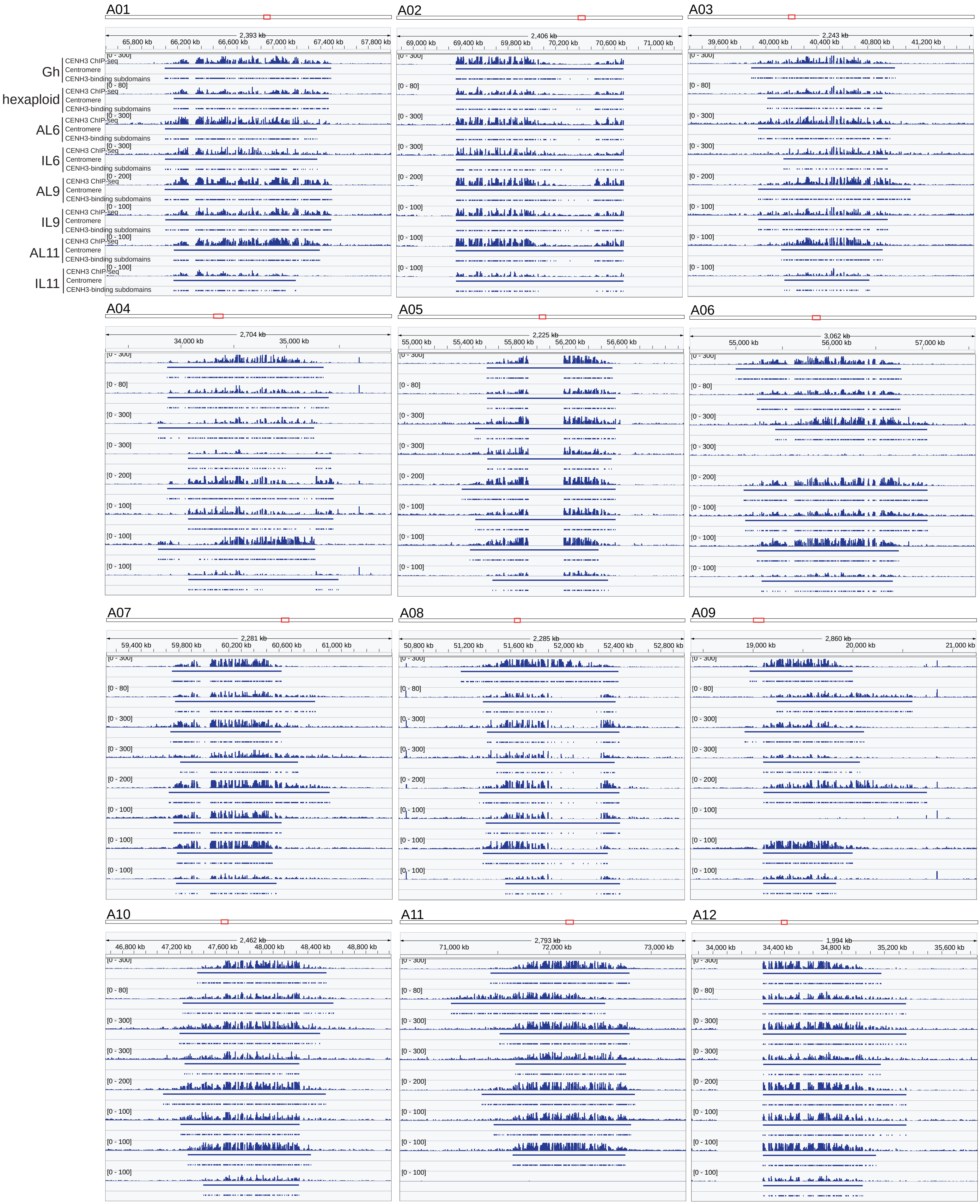

A01
2,393 kb
65,800 kb
66,200 kb
66,600 kb
67,000 kb
67,400 kb
67,800 kb
A02
2,406 kb
69,000 kb
69,400 kb
69,800 kb
70,200 kb
70,600 kb
71,000 kb
A03
2,243 kb
39,600 kb
40,000 kb
40,400 kb
40,800 kb
41,200 kb
Gh hexaploid
AL6
IL6
AL9
IL9
AL11
IL11
CENH3 ChIP-seq
Centromere
CENH3-binding subdomains
[0 - 300]
[0 - 80]
[0 - 200]
[0 - 100]
A04
2,704 kb
34,000 kb
35,000 kb
A05
2,225 kb
55,000 kb
55,400 kb
55,800 kb
56,200 kb
56,600 kb
A06
3,062 kb
55,000 kb
56,000 kb
57,000 kb
A07
2,281 kb
59,400 kb
59,800 kb
60,200 kb
60,600 kb
61,000 kb
A08
2,285 kb
50,800 kb
51,200 kb
51,600 kb
52,000 kb
52,400 kb
52,800 kb
A09
2,860 kb
19,000 kb
20,000 kb
21,000 kb
A10
2,462 kb
46,800 kb
47,200 kb
47,600 kb
48,000 kb
48,400 kb
48,800 kb
A11
2,793 kb
71,000 kb
72,000 kb
73,000 kb
A12
1,994 kb
34,000 kb
34,400 kb
34,800 kb
35,200 kb
35,600 kb

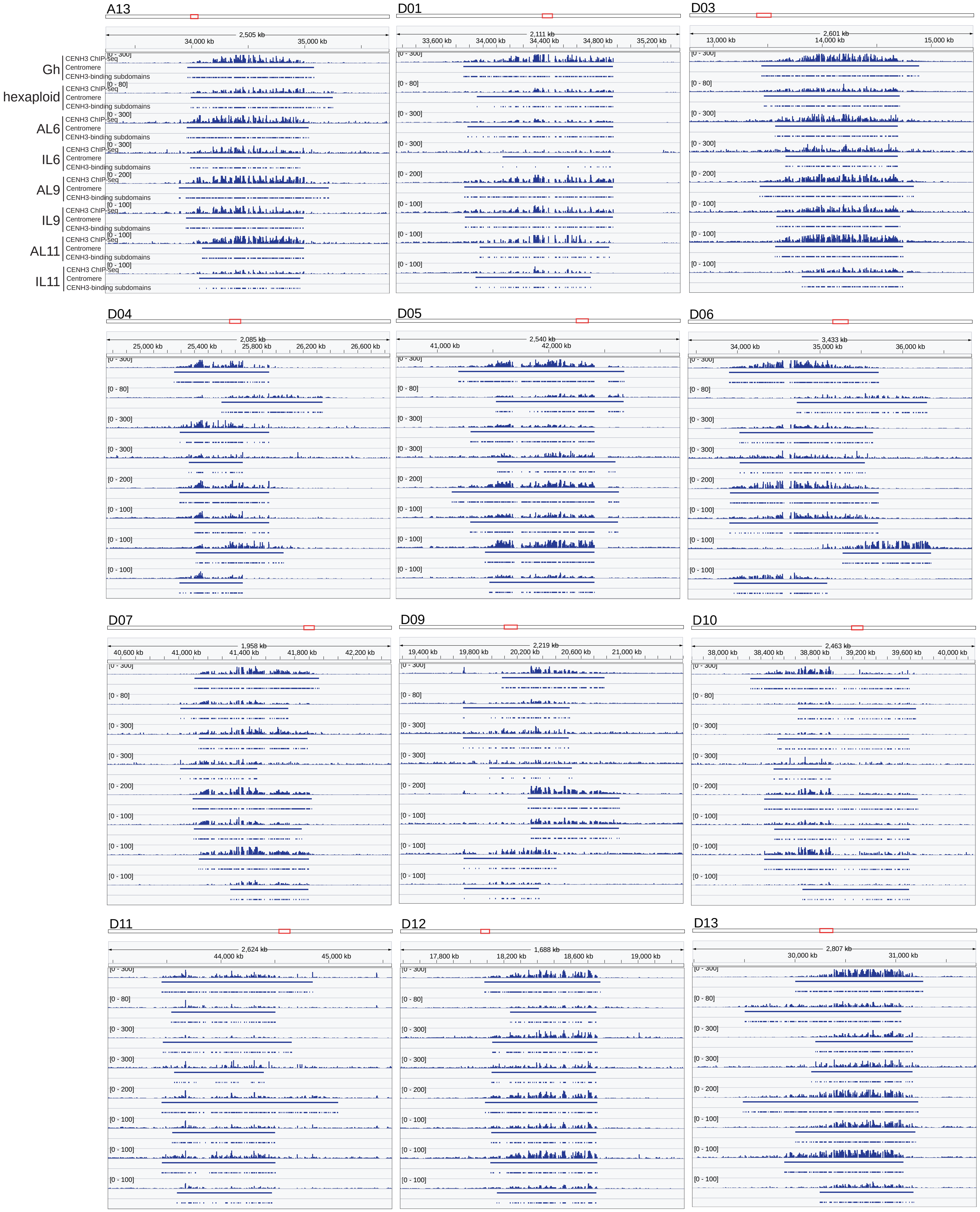

S29 Fig. IGV snapshots showing the centromeres and CENH3 binding subdomains on each chromosome of Gh.

The CENH3 ChIP-seq track displays the alignment of ChIP-seq reads, with bars in the centromere track indicating centromeric regions. The CENH3 binding subdomains track shows peaks identified by the MACS2 software, representing the CENH3 binding subdomains.
