# Supplementary material for: Centromere-size reduction and chromatin state dynamics following intergenomic hybridization in cotton
Source: PLoS Genet. 2025 May 2;21(5):e1011689. doi: 10.1371/journal.pgen.1011689 (PMC12068715; doi:10.1371/journal.pgen.1011689)
Supplement: S1 Table — (PDF) [file pgen.1011689.s030.pdf]

S1 Table. Summary of data generated in this study

Sample	Data type	Total reads	Mapped reads	Unique reads
Ga	CENH3 ChIP-seq	36138964	33353656	24746214
	Input-seq	45158322	40270729	25184634
AL9	CENH3 ChIP-seq	59116560	54224608	14719906
	Input-seq	67315078	65949995	43296544
IL9	CENH3 ChIP-seq	96339666	85467254	32268948
	Input-seq	55854156	53299217	28753650
AL6	CENH3 ChIP-seq	12512370	11802667	5423958
	Input-seq	18511676	18070173	10952960
IL6	CENH3 ChIP-seq	16965488	15105618	5126618
	Input-seq	15596210	14623492	6078318
AL11	CENH3 ChIP-seq	38279392	36748517	21024860
	Input-seq	40138022	35411849	21138726
IL11	CENH3 ChIP-seq	84707372	79580208	22598396
	Input-seq	43831714	39167499	23142640
hexaploid	CENH3 ChIP-seq	63996466	59256176	33983000
	Input-seq	35636086	31823387	15587704
Ga	RNA-seq Rep1	28475942	23808076	22760380
	RNA-seq Rep2	28230166	23374196	22318042
AL9	RNA-seq Rep1	66662916	55283336	50629882
	RNA-seq Rep2	72621608	60835168	55177792
IL9	RNA-seq Rep1	84679456	73133202	67776420
	RNA-seq Rep2	73839590	62726412	58120022
AL6	RNA-seq Rep1	75108712	63182932	57501782
	RNA-seq Rep2	73890692	62007308	56167060
IL6	RNA-seq Rep1	103003468	89084906	82215170
	RNA-seq Rep2	83795076	72865424	67322916
AL11	RNA-seq Rep1	79832556	66639490	60743564
	RNA-seq Rep2	71778186	60627454	55652104
IL11	RNA-seq Rep1	72713948	63133698	58107758
	RNA-seq Rep2	70287726	61370086	56477544
Ga	H3K4me3 ChIP-seq Rep1	34236536	32494765	26832370
	H3K4me3 ChIP-seq Rep2	53282334	50264431	42088462
	H3K27me3 ChIP-seq Rep1	61517010	44608431	40644818
	H3K27me3 ChIP-seq Rep2	58478966	42123717	38468854
	H3K36me3 ChIP-seq Rep1	48652460	46420665	38997610
	H3K36me3 ChIP-seq Rep2	52061070	49651771	41476284
	H3K27ac ChIP-seq Rep1	52126734	48870578	39027148
	H3K27ac ChIP-seq Rep2	46872240	43977995	34569666
	H3K9ac ChIP-seq Rep1	57331586	53021823	40223756
	H3K9ac ChIP-seq Rep2	45180492	41794094	30919018
	H3K9me3 ChIP-seq Rep1	37296926	35558953	30131612

	H3K9me3 ChIP-seq Rep2	42167182	40836971	36259858
	H3K4me1 ChIP-seq Rep1	60728670	59940425	54365716
	H3K4me1 ChIP-seq Rep2	67365046	66546247	60706844
	H3K27me2 ChIP-seq Rep1	68952942	60736690	45822408
	H3K27me2 ChIP-seq Rep2	65268930	57449513	43695888
	H3K36me2 ChIP-seq Rep1	79485708	77564754	69888496
	H3K36me2 ChIP-seq Rep2	64504006	62896176	56506486
	H3K4me2 ChIP-seq Rep1	55910078	55155323	50242238
	H3K4me2 ChIP-seq Rep2	55914484	55208688	50124964
AL9	H3K4me3 ChIP-seq Rep1	50833928	49709605	36424100
	H3K4me3 ChIP-seq Rep2	58586772	57415631	42305400
	H3K27me3 ChIP-seq Rep1	57685400	55619087	38425956
	H3K27me3 ChIP-seq Rep2	39576182	38382384	27799648
	H3K36me3 ChIP-seq Rep1	84658740	83864184	62960346
	H3K36me3 ChIP-seq Rep2	71409876	70702483	52589776
	H3K27ac ChIP-seq Rep1	63499566	62135746	45643670
	H3K27ac ChIP-seq Rep2	50093166	49007112	35930924
	H3K9ac ChIP-seq Rep1	54338896	52934951	37713838
	H3K9ac ChIP-seq Rep2	61230180	60053793	44734010
	H3K9me3 ChIP-seq Rep1	165152192	160109725	108597874
	H3K9me3 ChIP-seq Rep2	83266926	81509036	57597804
	H3K4me1 ChIP-seq Rep1	52166298	51552902	38769290
	H3K4me1 ChIP-seq Rep2	45335086	44670473	30288890
	H3K27me2 ChIP-seq Rep1	75769766	73321610	50696586
	H3K27me2 ChIP-seq Rep2	64491088	62340132	43039872
	H3K36me2 ChIP-seq Rep1	75095620	74050710	55315626
	H3K36me2 ChIP-seq Rep2	71972806	71018717	53643898
	H3K4me2 ChIP-seq Rep1	69238436	68771716	51080314
	H3K4me2 ChIP-seq Rep2	70328646	69616251	51853542
IL9	H3K4me3 ChIP-seq Rep1	43941476	41822686	29867212
	H3K4me3 ChIP-seq Rep2	41293556	39010836	28644384
	H3K27me3 ChIP-seq Rep1	50018594	48243218	33839472
	H3K27me3 ChIP-seq Rep2	65596942	63640201	47283174
	H3K36me3 ChIP-seq Rep1	54537414	53823335	41133518
	H3K36me3 ChIP-seq Rep2	63989046	63149773	48646878
	H3K27ac ChIP-seq Rep1	48491692	47091254	33360792
	H3K27ac ChIP-seq Rep2	55588042	53964535	37857518
	H3K9ac ChIP-seq Rep1	58633028	57241861	40263184
	H3K9ac ChIP-seq Rep2	69496430	67787149	47751080
	H3K9me3 ChIP-seq Rep1	56978822	55437548	39673246
	H3K9me3 ChIP-seq Rep2	53395414	51951516	37408872
	H3K4me1 ChIP-seq Rep1	47696412	47085293	35938044
	H3K4me1 ChIP-seq Rep2	54117756	53437717	40384080
	H3K27me2 ChIP-seq Rep1	47270596	45342092	27500182
	H3K27me2 ChIP-seq Rep2	71443230	68510406	43096926
	H3K36me2 ChIP-seq Rep1	61350998	60437257	46066350

	H3K36me2 ChIP-seq Rep2	73120606	71988882	54562750
	H3K4me2 ChIP-seq Rep1	62978798	62265152	44917952
	H3K4me2 ChIP-seq Rep2	61412416	60725833	43712612
hexaploid	H3K27ac ChIP-seq Rep1	103246944	91158770	61945946
	H3K27ac ChIP-seq Rep2	108364512	94926745	64694176
	H3K36me2 ChIP-seq Rep1	113628918	109200117	81832234
	H3K36me2 ChIP-seq Rep2	106933942	102863818	76717518
	H3K27me3 ChIP-seq Rep1	159531220	148921514	104234352
	H3K27me3 ChIP-seq Rep2	158993030	149868187	109906528
	H3K4me2 ChIP-seq Rep1	172382814	165818239	116892682
	H3K4me2 ChIP-seq Rep2	141722414	136376228	95879522
Ga	DNase-seq Rep1	138148554	132259802	104054698
	DNase-seq Rep2	127894510	123957516	100261274
AL9	DNase-seq Rep1	59966660	58024336	30945872
	DNase-seq Rep2	71258834	68578438	39110118
IL9	DNase-seq Rep1	48617282	46653102	28427164
	DNase-seq Rep2	69346718	66063634	40017972

**Sample**	**Data type**	**Sequenced read pairs**	**Pairs mappable**	**Valid read pairs (Hi-C contacts)**
Ga	Hi-C Rep1	672343919	429522140	142241599
	Hi-C Rep2	714284707	474395328	290417457
IL9	Hi-C Rep1	674117225	366959642	226996859
	Hi-C Rep2	651388711	358540266	177326946
