# Supplementary material for: Centromere-size reduction and chromatin state dynamics following intergenomic hybridization in cotton
Source: PLoS Genet. 2025 May 2;21(5):e1011689. doi: 10.1371/journal.pgen.1011689 (PMC12068715; doi:10.1371/journal.pgen.1011689)
Supplement: S2 Table — (PDF) [file pgen.1011689.s031.pdf]

S2 Table. Centromere positions for each chromosome of *G. anomalum*

Chr.	*G. anomalum*		hexaploid	
	Position (Mb)	Length (Mb)	Position (Mb)	Length (Mb)
Chr01	56.59-57.91	1.32	56.64-57.80	1.16
Chr02	35.79-36.67	0.88	35.97-36.68	0.71
Chr03	54.88-55.96	1.08	54.92-55.82	0.90
Chr04	45.62-47.13	1.51	46.72-47.10	0.38
Chr05	52.82-53.92	1.10	53.17-53.88	0.71
Chr06	52.54-53.83	1.29	52.60-53.71	1.11
Chr07	59.13-60.12	0.99	59.26-59.98	0.72
Chr08	43.72-44.84	1.12	43.82-44.60	0.78
Chr09	45.67-46.71	1.04	46.06-46.68	0.62
Chr10	60.63-62.01	1.38	60.85-61.64	0.79
Chr11	57.81-58.85	1.04	57.82-58.41	0.59
Chr12	39.18-40.03	0.85	39.19-40.02	0.83
Chr13	48.31-48.77	0.46	48.44-48.55	0.11
